# A genome-wide One Health study of *Klebsiella pneumoniae* in Norway reveals overlapping populations but few recent transmission events across reservoirs

**DOI:** 10.1186/s13073-025-01466-0

**Published:** 2025-04-28

**Authors:** Marit A. K. Hetland, Mia A. Winkler, Håkon P. Kaspersen, Fredrik Håkonsholm, Ragna-Johanne Bakksjø, Eva Bernhoff, Jose F. Delgado-Blas, Sylvain Brisse, Annapaula Correia, Aasmund Fostervold, Margaret M. C. Lam, Bjørn-Tore Lunestad, Nachiket P. Marathe, Niclas Raffelsberger, Ørjan Samuelsen, Marianne Sunde, Arnfinn Sundsfjord, Anne Margrete Urdahl, Ryan R. Wick, Iren H. Löhr, Kathryn E. Holt

**Affiliations:** 1 Department of Medical Microbiology, Stavanger University Hospital, Stavanger, Norway; 2 Department of Biological Sciences, Faculty of Science and Technology, University of Bergen, Bergen, Norway; 3 Department of Medical Biology, Faculty of Health Sciences, UiT the Arctic University of Norway, Tromsø, Norway; 4 Research Section Food Safety and Animal Health, Department of Animal Health and Food Safety, Norwegian Veterinary Institute, Ås, Norway; 5 Institute of Marine Research, Bergen, Norway; 6 Biodiversity and Epidemiology of Bacterial Pathogens Unit, Institut Pasteur, Université Paris Cité, Paris, France; 7 Department of Infection Biology, Faculty of Infectious and Tropical Diseases, London, School of Hygiene & Tropical Medicine, London, UK; 8 Department of Clinical Science, Faculty of Medicine, University of Bergen, Bergen, Norway; 9 Department of Infectious Diseases, School of Translational Medicine, Monash University, Melbourne, Australia; 10 Department of Microbiology and Infection Control, University Hospital of North Norway, Tromsø, Norway; 11 Norwegian National Advisory Unit on Detection of Antimicrobial Resistance, Department of Microbiology and Infection Control, University Hospital of North Norway, Tromsø, Norway; 12 Section for Bacteriology, Department for Analysis and Diagnostics, Norwegian Veterinary Institute, Ås, Norway; 13 Department of Microbiology and Immunology, University of Melbourne at the Peter Doherty Institute for Infection and Immunity, Melbourne, Australia

**Keywords:** One Health, *Klebsiella pneumoniae* species complex, Genomics, GWAS, AMR, Transmission, Zoonotic transmission, Ecology

## Abstract

**Background:**

Members of the *Klebsiella pneumoniae* species complex (KpSC) are opportunistic pathogens that cause severe and difficult-to-treat infections. KpSC are common in non-human niches, but the clinical relevance of these populations is disputed.

**Methods:**

In this study, we analysed 3255 whole-genome sequenced isolates from human, animal and marine sources collected in Norway between 2001 and 2020. We used population genomics in a One Health context to assess the diversity of strains, genes and other clinically relevant genetic features within and between sources. We further explored niche-enriched traits using genome-wide association studies and investigated evidence of spillover and connectivity across the KpSC populations from the three niches.

**Results:**

We found that the KpSC populations in different niches were distinct but overlapping. Overall, there was high genetic diversity both between and within sources, with nearly half (49%) of the genes in the accessory genome overlapping the ecological niches. Further, several sublineages (SLs) including SL17, SL35, SL37, SL45, SL107 and SL3010 were common across sources. There were few niche-enriched traits, except for aerobactin-encoding plasmids and the bacteriocin colicin a, which were associated with KpSC from animal sources. Human infection isolates showed the greatest connectivity with each other, followed by isolates from human carriage, pigs, and bivalves. Nearly 5% of human infection isolates had close relatives (≤22 substitutions) amongst animal and marine isolates, despite temporally and geographically distant sampling of these sources. There were limited but notable recent spillover events, including the movement of plasmids encoding the virulence locus *iuc*3 between pigs and humans.

**Conclusions:**

Our large One Health genomic study highlights that human-to-human transmission of KpSC is more common than transmission between ecological niches. Still, spillover of clinically relevant strains and genetic features between human and non-human sources does occur and should not be overlooked. Infection prevention measures are essential to limit transmission within human clinical settings and reduce infections. However, preventing transmission that leads to colonisation, e.g. from direct contact with animals or via the food chain, could also play an important role in reducing the KpSC disease burden.

**Supplementary Information:**

The online version contains supplementary material available at 10.1186/s13073-025-01466-0.

## Background

*Klebsiella pneumoniae* are a frequent cause of difficult-to-treat multidrug-resistant (MDR) infections. Additionally, strains with acquired virulence factors can cause severe hypervirulent infections, and convergent strains displaying both MDR and hypervirulent traits are rapidly emerging [1]. The *K. pneumoniae* species complex (KpSC) consists of seven species/subspecies that can be further divided into sublineages (SLs), comprising groups of closely related core-genome sequence types (STs) [2–4]. The KpSC are opportunistic pathogens that often colonise people before subsequently causing infections [5]. They can also be found in environmental sources, including terrestrial and marine animals and food, which could provide a source of transmission to humans [6, 7]. However, it has been unclear to what extent KpSC populations isolated from humans are distinct from those found in other niches, and whether non-human sources serve as reservoirs for clinically relevant strains, contributing to the pool of bacteria that cause colonisation and infection in humans. For example, depending on the availability of nutrients and exposure to substances such as antibiotics and heavy metals, KpSC from different niches may display distinct metabolic traits and antimicrobial resistance (AMR) levels due to selective pressures and niche adaptation [8, 9]. By identifying niche-specific traits, we may better understand the ecological distribution of strains, and distinguish which KpSC niches represent clinically relevant reservoirs of infections in humans versus those with little relevance to human health.

To study the emergence, dynamics and spread of KpSC within and between niches, a One Health approach can be applied, using whole-genome sequencing to characterise and compare bacterial isolates from diverse sources. A One Health perspective considers the health of people, animals and environments as a whole, recognising that changes in one can greatly affect another. Recognising the critical role of this approach, the Quadripartite Joint Secretariat on AMR has launched a global One Health Joint Action Plan, signalling its importance in addressing AMR on an international scale [10]. The results from such studies have the potential to guide public health interventions, by identifying routes of transmission that could be interrupted to reduce infections, or by identifying niches where selection for clinically relevant AMR or virulence determinants is occurring.

Only a few One Health studies of KpSC have previously been reported, and few (if any) have assessed niche-adaptation in KpSC*.* These largely concluded that short-term transmission of KpSC is more common within clinical settings than between clinical and other sources, although transmission to humans from other sources does occasionally occur [6, 11, 12]. This is in contrast to classic foodborne bacterial pathogens such as *Salmonella enterica* or *Campylobacter jejuni*, where human infections tend to result from short-chain transmission from animal reservoirs via consumption of contaminated food [13]. Still, even if KpSC spillover events occur only rarely, they can have significant impacts on human health. To take some well-known examples from virology, the COVID- 19 pandemic and MERS epidemic are thought to stem from very rare but high-impact zoonotic transmission from animals to humans, followed by sustained human-to-human transmission [14, 15]. Given that KpSC—unlike *S. enterica* or *C. jejuni*—can cause persistent colonisation of humans and can easily spread between humans, at least in healthcare settings, it is reasonable to propose that even occasional transmission of AMR or hypervirulent KpSC strains from animal reservoirs to humans could have sustained impacts on human health by introducing new clinically relevant strains that subsequently become established in humans.

Since the vast majority of publicly available KpSC genome sequences are derived from humans, there is insufficient data to ascertain whether any of the lineages that are common in humans have an animal origin. However, there are known examples of recent spillover of clinically relevant mobile genetic elements (MGEs) from animals to humans, which have since spread within human-associated KpSC populations. For instance, the *mcr-1* gene, which confers resistance to the last-line antibiotic colistin, is believed to have originated in animals and subsequently spilled over to humans, where it now contributes to difficult-to-treat KpSC and other Enterobacterales infections [16]. Another example is the pig-associated aerobactin virulence locus *iuc*3, which has been found in multiple KpSC lineages colonising humans and causing infections [17, 18]. It is therefore important to understand how clinically relevant KpSC strains and MGEs enter and circulate among people to inform and support public health intervention measures to prevent disease, health care burdens and mortality.

Investigation of transmission between niches requires sympatric sampling, which has been extremely limited for KpSC [6, 11, 19, 20]. Through national surveillance programs in the human and veterinary sectors, and screening of human community carriage, animals and marine samples, >3000 KpSC isolates from across Norway over 20 years have been collected and sequenced [17, 21–25]. The isolates were originally sampled to quantify the burden of infection or carriage in distinct niches, without bias towards AMR or hypervirulence phenotypes. Due to the low prevalence of AMR in Norway [26], this provides a unique opportunity to study niche connectivity in a population largely unaffected by antibiotic selection pressures. Here, we report on the diversity and niche-association of strains, genes and other clinically relevant genetic features across a collection of 3255 KpSC genomes from human, animal and marine niches, and explore evidence for the separation and connectivity of KpSC populations isolated from these niches.

## Methods

### Sample selection and whole-genome sequencing

We included 3255 KpSC isolates collected between 2001 and 2020 in this study. Isolates from human infections (*n* = 1920 blood; 252 urine) were collected as part of two nationwide surveillance studies covering all 22 clinical microbiology laboratories across Norway, between 2001 and 2018 [21, 25]. Human faecal carriage isolates (*n* = 484) were collected from a general adult population in the Tromsø municipality in Norway during 2015–2016 [22]. From marine environments, KpSC isolates (*n* = 99) were recovered from 7 surface seawater samples and 92 bivalves and sea urchins (in 2016, 2019 and 2020), but none were recovered from fish or sediment [24, 27]. The samples were collected from across the Norwegian coast, from 77 production areas and 5 non-rearing locations. For bivalves and sea urchins, batches of 10–20 individuals were pooled to obtain an isolate. The samples from fish, surface seawater and sediment were from unique individuals or samples. The animal samples were collected from farms across the country, to represent the Norwegian populations of the sampled animals. Carriage (caecal or faecal) samples from pigs (*n* = 146, collected in 2019), turkey flocks (*n* = 113 in 2018, *n* = 60 in 2020), broiler flocks (*n* = 90 in 2018 and *n* = 55 in 2020), wild boars (*n* = 27 in 2020), dogs (*n* = 16 in 2019) and cattle (*n* = 12 in 2019) were collected via the Norwegian monitoring program for AMR in the veterinary sector (NORM-VET) [17, 23, 28]. From turkeys and broilers, ten caecal samples were pooled from each flock to obtain one sample for screening. From pigs, dogs and cattle, only one animal was sampled per herd. For wild boars, individual animals were sampled. Additionally, 20 infection isolates were included from individual dogs, turkeys and broilers. Although KpSC carriage rates for cattle and wild boar are reported, their isolates were not whole-genome sequenced and were excluded from further analyses. Not all KpSC-positive carriage isolates from the turkey and broiler flocks were whole-genome sequenced, however, those that were included covered the whole sampling year. The dataset was divided into eight source types for comparison throughout the study: KpSC from human infections, community carriage, dogs, pigs, turkeys, broilers, marine bivalves and seawater.

The isolates were short-read sequenced using the Illumina MiSeq (*n* = 3218) or HiSeq 2500 (*n* = 37) platforms as described previously [21–23, 27], adapter- and quality filtered with TrimGalore v0.6.7 [29] and assembled with SPAdes v3.15.4 [30]. We additionally performed Oxford Nanopore Technologies long-read sequencing of 16.9% (*n* = 550/3255) of the collection (see Additional file 1: Supplementary Methods for selection approach), to produce closed hybrid genomes, as described in [31]. All genomes were quality controlled as described in Additional file 1: Supplementary Methods and in Additional file 2: Table S1.

### Genotyping and annotation

Kleborate v2.4.0 [32] was used to identify species, STs, virulence and AMR genes. K and O loci were identified with Kaptive v2.0.8 [33]. The presence of the *rmpADC* locus (with/without truncated *rmpA*) was used to define a likely hypermucoid phenotype [34]. Plasmid replicon markers were identified with plasmidfinder-db v2023-01-18 [35] using abricate v1.0.1 [36]. Life identification number (LIN) codes based on core genome multilocus sequence type (cgMLST) profiles were assigned to define and name KpSC SLs according to the 3-level LIN code prefixes [4], using the *Klebsiella* BIGSdb-Pasteur web tool [37]. This approach allows for more precise classifications than STs, as cgMLST-based LIN codes have been shown to be highly concordant with phylogenetic relationships [4]. For most isolates, the ST and SL identifiers align, as described in [2] and shown in Additional file 2: Table S1. SLs were compared to globally prevalent clonal groups (CGs) [1] with the same number, except for SL17 which was considered equivalent to CG20. Bakta v1.8.1 [38] with database v5.0 was used to annotate all assembled genomes, using the complete flag for circularised hybrid-assembled genomes and otherwise default settings. Bakta was run with NCBI’s AMRFinder database v2023-04-17.1, from which heavy metal- and thermoresistance genes were identified (see Additional file 1: Supplementary Methods). The genotyping results are available in Additional file 2: Table S1.

### Assessing niche enrichment

To assess differences in genetic diversity between the three ecological niches, and enrichment of genetic features within each niche, we first estimated the pangenome with panaroo v1.3.3 using the strict mode [39], using the annotations from Bakta as input. Panstripe v0.1.0 [40] was then used to compare gene gain and loss rates between the niches. To assess niche-enrichment of genes, unitigs and single nucleotide polymorphisms (SNPs), we performed genome-wide association studies (GWAS) with pyseer v1.3.11 [41] as described in Additional file 1: Supplementary Methods.

### Inferring cross-talk between niches

There were 107 SLs represented by ≥ 1 genome in ≥ 2 ecological niches. To assess the relatedness of strains within these SLs across the niches, we created reference genomes for each and ran whole-genome SNP alignments using RedDog v1beta.11 [42], as described previously [43], aligning all short-read genomes belonging to that SL against the reference genome (overall mean chromosomal coverage was 96.9% (range 88.6–100%), Additional file 2: Table S2). Pairwise SNP distances were identified from the alignments with snp-dists v0.8.2 [44]. We identified an optimal cutoff-point of ≤ 22 SNPs using cutpointR v1.1.2 [45] (see Additional file 3: Fig. S1). To record strain-sharing events, networks were created using a similar method as in [6]: using a SNP threshold of ≤ 22 SNPs, and counting each source-pair (including same-source pairs) only once per cluster. We also inferred dated phylogenies of SLs that had ≥ 20 genomes and were sampled over at least a 5-year period, to ensure sufficient temporal signal (Additional file 2: Table S3 and S4); in total 15 SLs, of which eight converged and had significant clock signal. Verticall distance v0.4.2 [46] was used to filter recombinations and BactDate v1.1.1 [47] to infer the phylogenies (see Additional file 1: Supplementary Methods).

### Definitions

Multidrug resistance was defined based on the presence of AMR genes or mutations identified by Kleborate v2.4.0: num_resistance_classes count ≥ 3.

### Statistical analyses

Statistical analyses were performed with R version 4.4.0 (2024–04–24). Comparisons of proportions were analysed with chi-squared tests, ranges with Kruskal–Wallis test (for overall groups) and Mann–Whitney *U* test (for each pair of groups), and distributions with Kolmogorov–Smirnov test. Binomial tests with Bonferroni correction were used to test whether features found in humans or non-human sources were overrepresented in the other. All tests were two-tailed. *P*-values < 0.05 from these tests were considered statistically significant, and significance was reported as follows: * *P* < 0.05, ** *P* < 0.01, *** *P* < 0.001, ns *P* ≥ 0.05. Significance thresholds for the GWAS results were decided by inspecting QQ-plots (Additional file 3: Fig. S2).

## Results

### KpSC were present among all sources collected on land and in coastal waters

**Fig. 1:**
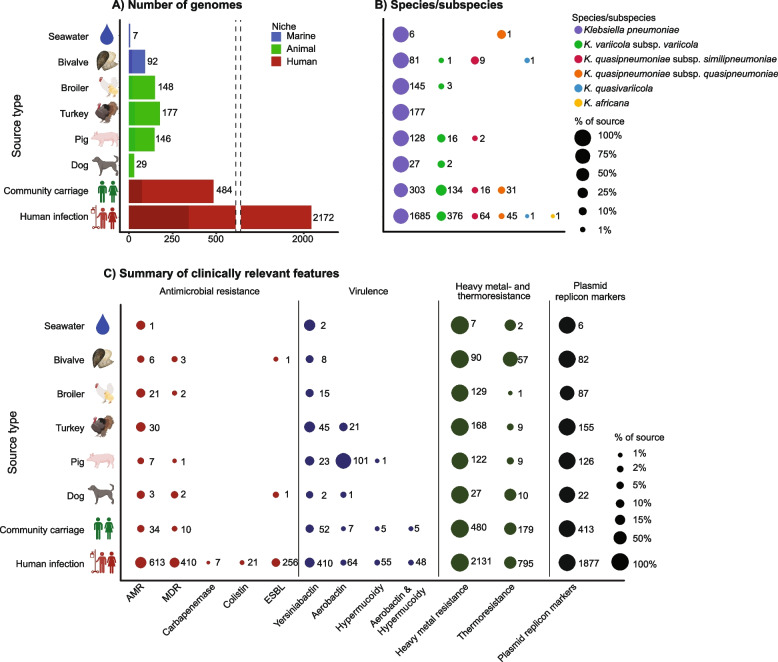
Key characteristics of the 3255 *Klebsiella pneumoniae* species complex (KpSC) isolates.** A** Distribution of genomes by source, with bars coloured by niche (inset legend), and shaded to indicate sequencing type (lighter for short-read only, darker for short- and long-read). Note that the *x*-axis is broken due to the large number of genomes from human infections. **B** KpSC species (inset legend) distribution by source. **C** Summary of clinically relevant features, showing the presence (bubble) of antimicrobial resistance determinants, virulence determinants, heavy metal- and thermoresistance operons/genes, and plasmid replicon markers across the sources. In **B** and **C**, the bubble size corresponds to the percentage of isolates with these features, with the number of isolates shown beside each bubble. *Abbreviations*: *AMR* Antimicrobial resistance, *MDR* multidrug resistance

We analysed 3255 KpSC genomes in this study (Table 1), which, with the exception of human carriage isolates, were collected to represent nationwide sampling. Of those, 2172 (66.7%) were from human infections (*n* = 1920 blood; 252 urine) from nationwide surveillance studies covering all 22 Norwegian clinical microbiology laboratories between 2001 and 2018 [21, 25]. Twenty genomes (0.6%) were from animal infections (2018–2020; 13 dog, 4 turkey, 3 broiler). Isolates from 2015 to 2020 from human and animal carriage studies and from marine samples in Norway were also included (*n* = 1063, 32.7%). The human community carriage samples were collected from a general adult population in the Tromsø municipality. The animal and marine samples were collected to represent the populations across Norway and the Norwegian coast, respectively (see Methods for sampling details). As previously reported, carriage rates estimated in these studies were 16% in humans [22] and 0–69% in different animals, with the highest rates in poultry flocks and pigs (Table 1) [17, 23, 24]. In addition, KpSC were found in 41.2% (7/17) of surface seawater samples. All isolates were short-read sequenced and 16.9% (*n* = 550) were additionally long-read sequenced. The genomes were divided into three ecological niches (human, animal and marine) and eight source types for comparisons (Fig. 1A).

**Table 1:** *Klebsiella pneumoniae* species complex (KpSC) infection and carriage in Norway

Host (infection status)	Collection years	KpSC positive carriage (%)/number of samples tested *	KpSC genomes included in this study **
**Human niche**			
Blood (infection) [21, 25]	2001, 2004–2018	NA	1920
Urine (infection) [21]	2003, 2009, 2012–2015	NA	252
Community carriage (carriage) [22]	2015, 2016	484 (16.3%)/2975	484
**Animal niche**			
Turkey flocks (carriage) [23, 28]	2018, 2020	191 (69.2%)/276	173
Turkeys (infection) [23, 28]	2018, 2020	NA	4
Broiler flocks (carriage) [23, 28]	2018, 2020	158 (25.2%)/627	145
Broilers (infection) [23, 28]	2018, 2019	NA	3
Dogs (carriage)	2019	16 (6.5%)/246	16
Dogs (infection)	2018–2020	NA	13
Pigs (carriage) [17]	2019	146 (48.8%)/299	146
Cattle (carriage)	2019	12 (4.0%)/299	0
Wild boars (carriage)	2020	27 (21.4%)/126	0
Marine niche ***			
Bivalves [24]	2016, 2019, 2020	92 (15.9%)/578	92
Surface seawater [24]	2019	7 (41.2%)/17	7
Sea urchin	2019	0 (0%)/7	0
Fish	2019, 2020	0 (0%)/53	0
Sediment	2019	0 (0%)/24	0
**Total**		1133 (20.5%)/5527	3255

*KpSC* *Klebsiella pneumoniae* species complex, *NA* Not applicable

*Some of the animal samples were pooled before assessing KpSC presence; see Methods for sampling details

**Not all KpSC-positive isolates from turkey and broiler flocks were whole-genome sequenced, however, those included covered the whole sampling year

***Infection status was not available for the marine samples. References are provided in the table for the sources whose carriage rates or isolates were previously reported

*K. pneumoniae* is the major species within the KpSC in clinical settings [1]. In our collection, it was the most common species within all sources ranging from 62.6 to 100% (Fig. 1B). The 3255 genomes belonged to 857 SLs (Additional file 2: Table S2), the majority of which were represented by only a few genomes each: 87.8% (752/857) by ≤ 5 genomes; 59.0% (506/857) were singletons. Simpson’s diversity indices of SLs were 0.99 for the human, 0.98 for animal and 0.96 for marine niche. Despite high diversity, there was considerable overlap between the niches: 12.5% (107/857) were found in at least two niches and accounted for nearly half (48.5%, 1578/3255) of the genomes. These SLs belonged to *K. pneumoniae* (*n* = 91), *K. variicola* subsp. *variicola* (*n* = 12) and *K. quasipneumoniae* subsp. *similipneumoniae* (*n* = 4).

### AMR was concentrated in human KpSC isolates

We observed low levels of acquired AMR, reflecting the low use of antibiotics in Norway in humans and animals [26]. Still, AMR determinants were much more common in humans (24.4% with acquired AMR, 15.8% MDR, 9.6% extended-spectrum β-lactamase (ESBL)) than in animals (12.2% AMR, 1.0% MDR, 0.2% ESBL) and marine sources (7.1% AMR, 3.0% MDR, 1.0% ESBL) (*P* < 0.001, see Additional file 2: Table S5, Fig. 1C, Additional file 3: Figs. S3 and S4). Only two isolates from non-human sources encoded ESBLs: SL1035 with *bla*_CTX-M-3_ (from a bivalve), and SL1583 with *bla*_CTX-M-15_ (from a canine infection). Neither the strains nor plasmids from these isolates were present elsewhere in our collection.

AMR determinants were distributed across SLs, found among 24.4% (189/776) of SLs in isolates from humans, 16.2% (24/148) in animals and 13.5% (7/52) in the marine niche. Of eight recognised MDR-associated clones (SL101, SL147, SL15, SL258, SL29, SL307, SL37 and SL17) [1], all were found among the human infection isolates. SL17, SL37 and SL29 were also seen in community carriage, animal and marine isolates, and one SL258 isolate in community carriage. Of 488 genomes with these eight SLs, only 34.8% (*n* = 170) were MDR and 25.8% (*n* = 126) encoded ESBLs or carbapenemases. The isolates in these SLs in the community carriage, animal and marine isolates were not MDR (except one marine SL37) and did not encode any ESBLs or carbapenemases (Additional file 3: Fig. S5A).

### Animals as reservoirs for virulence factors

Known acquired virulence factors were not as concentrated in the human niche as AMR determinants (Fig. 1C, Additional file 3: S4 and S6). There were no significant differences in yersiniabactin prevalence across the niches, ranging from 6.9 to 28.6% per source. Aerobactin-encoding loci were present in 6.0% (194/3255) of genomes, the majority in animals: 10.8% (21/194) belonged to a clonal expansion of an SL290 strain that carried an IncFIB/IncFII plasmid encoding *iuc*5 and *iro*5 in turkey isolates, described previously [23]; neither the chromosome nor plasmid were seen elsewhere in our dataset. Accounting for 52.1% of the aerobactin-encoding genomes, IncFIB(K)/IncFII *iuc*3-encoding plasmids (and one chromosome) were seen in 101 pig isolates, in 50 SLs, as previously reported [17]*. iuc*3 was rare in human isolates (8 infections, 2 carriage). A comparison with publicly available *iuc*3-encoding genomes [18] showed that two of these had close relatives with the Norwegian pig isolates: one community carriage and one pig isolate were clonally related (SL10332, 0 *iuc*3 plasmid SNPs, 100% replicon sequence coverage), the other pair belonged to SL35 (human blood) and SL10334 (pig) and shared 4 *iuc*3 plasmid SNPs and 99.9% *iuc*3 plasmid coverage, consistent with local sharing between humans and pigs (e.g. via food or direct contact with pigs). The remaining *iuc*3-encoding plasmids in humans had no local relatives amongst human or pig isolates in our collection (> 20 SNPs), but were related to human clinical *iuc*3 isolates from Asia or Europe (closest pairwise relatives shared 1–46 SNPs, median 12.5), as previously noted [18], which may reflect imported strains (e.g. via imported food or human travel).

Hypervirulence-associated clones [1] were detected only in the human niche (SL23, SL25, SL380, SL66 and SL86), except for one SL25 genome (*ybt*6; ICE*Kp5*, K locus KL2 and MDR) in the marine niche which was closely related to an isolate from the human niche (see Additional file 3: Fig. S5B and below). The genomes belonging to these clones had capsule locus KL1 or KL2 and carried aerobactin loci *iuc*1 or *iuc*2, salmochelin *iro*1 or *iro*2 and the hypermucoidy loci *rmp*1 or *rmp*2 (except 44/45 SL25 genomes that encoded no *iuc*, *iro* or *rmp* loci).

### Heavy metal and thermoresistance associated with human and marine sources

**Fig. 2:**
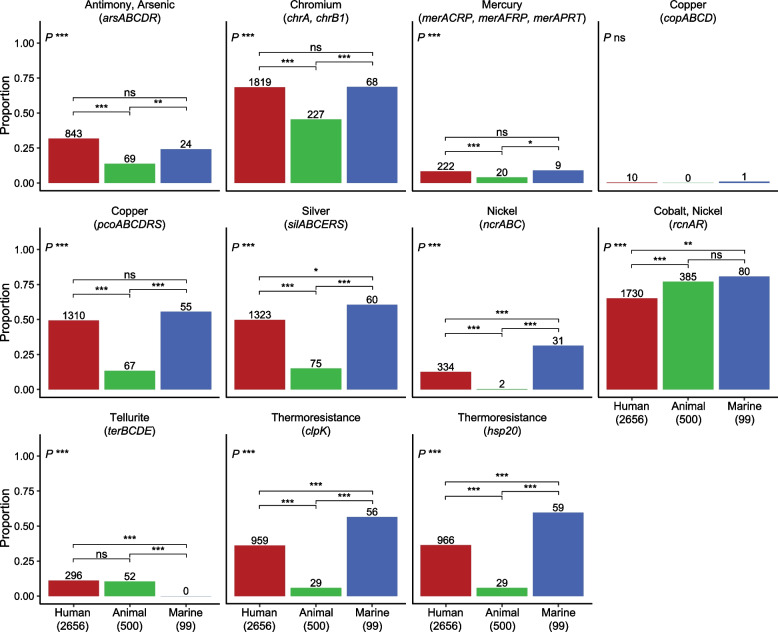
Heavy metal- and thermoresistance by niche. The proportion of genomes in the human, animal and marine niches that carried heavy metal resistance operons and thermoresistance genes. Statistical comparisons were performed with Chi-squared tests. Significance levels are indicated in the plot as follows: * *P* < 0.05, ** *P* < 0.01, *** *P* < 0.001, ns *P* ≥ 0.05

Heavy metal and thermoresistance loci, which may facilitate survival and/or maintenance of antibiotic resistance mechanisms, were present in all niches (Fig. 1C), but the frequencies of different operons varied significantly between niches (Fig. 2). The most common heavy metal resistances for all niches were to chromium and cobalt/nickel (typically chromosomally encoded; Additional file 3: Figs. S7 and S8). However, these showed distinct niche distributions, with *chrA/B1* (chromium resistance) significantly less prevalent in animal isolates (45.4% vs 68.5% in humans, *P* < 0.001) and *rcnAR* (cobalt/nickel resistance) less prevalent in human isolates (65.1% vs 77% in animals, *P* < 0.001). Silver (*sil*), copper (*pco*), arsenic (*ars*) and thermoresistance (*clpK*, *hsp20*) operons/genes were also more prevalent in human and marine isolates (> 25%) compared to animals (≤ 15%; *P* < 0.001, see Fig. 2), and were typically plasmid-encoded (Additional file 3: Fig. S7). We note a correlation between the two most common plasmid replicon markers (IncFIB(K) and IncFII(pKP91)) and genes encoding resistance to several antibiotic classes (aminoglycosides, sulfonamides, trimethoprim, ESBLs) as well as to heavy metals (*sil*, *pco*) and thermoresistance (Additional file 3: Fig. S9). In total, 93 genomes from the human niche carried all of these determinants (Additional file 3: Fig. S10), together with IncFIB and IncFII plasmid replicon markers. They belonged to 28 SLs, the most common were SL307 (*n* = 36), SL15 (*n* = 7), SL45 (*n* = 7), SL17 (*n* = 5), SL258 (*n* = 5) and SL35 (*n* = 5). Additionally, several genomes in all three niches carried these plasmid replicon markers, heavy metal and thermoresistance genes without the AMR determinants.

### Large pangenome overlap with few niche-enriched traits

**Fig. 3:**
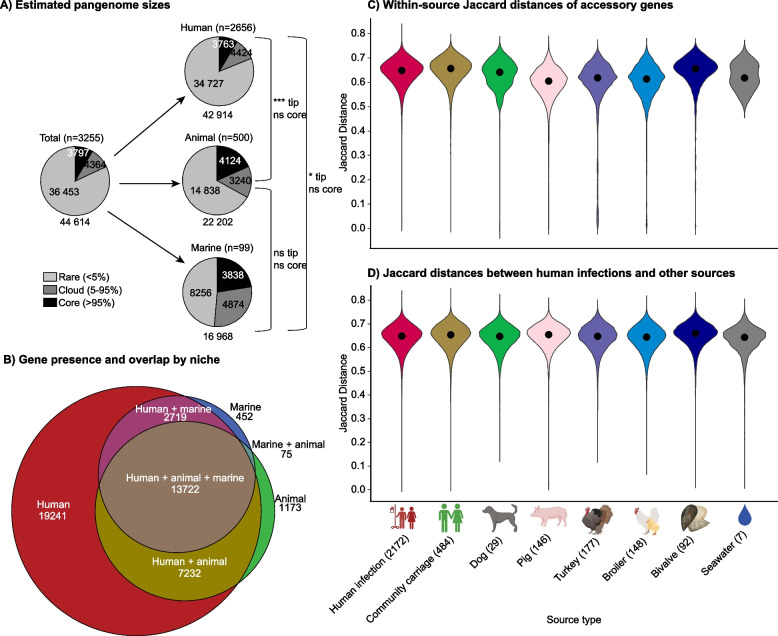
Comparison of the pangenome across niches.** A** Overall counts of core and accessory genes, in total and by niche. A panstripe analysis indicated no significant differences in gene gain or loss rates across the niches, but the human niche had a higher rate of rare genes observed at the tips of the phylogeny (tip; *P* < 0.001 when compared with animal genomes, and *P* < 0.05 compared with marine genomes), which could be driven by a combination of an increased number of highly mobile or singleton genes present in the human niche in addition to technical variation of the annotation algorithm. **B** Euler diagram showing presence and overlap of the 44,614 unique genes in the pangenome across the niches. **C** Pairwise Jaccard distances of accessory genes within each source. **D** Between-source pairwise Jaccard distances of accessory genes. For each source, the Jaccard distance of each genome was compared with the distance in human infection genomes to compare diversity between sources. In **C** and **D**, black points indicate median values

We next investigated the pangenome of our genome collection, which consisted of 44,614 unique genes, of which the majority (81.7%; *n* = 36,453) were rare (i.e. present in < 5% of genomes), 9.8% (*n* = 4364) were variably present (in 5–95% of genomes) and 8.5% (*n* = 3797) were core (in ≥ 95% of genomes). Nearly half of the accessory (< 95% of genomes) genes (48.9%; 19,951/40,817) were niche-overlapping. The human niche had a much larger accessory genome, which was expected due to the larger sample size and collection time. However, the gene content diversity and gain/loss rates were similar across the niches, as estimated by comparing Jaccard distances and gene gain/loss rates (Fig. 3).

**Fig. 4:**
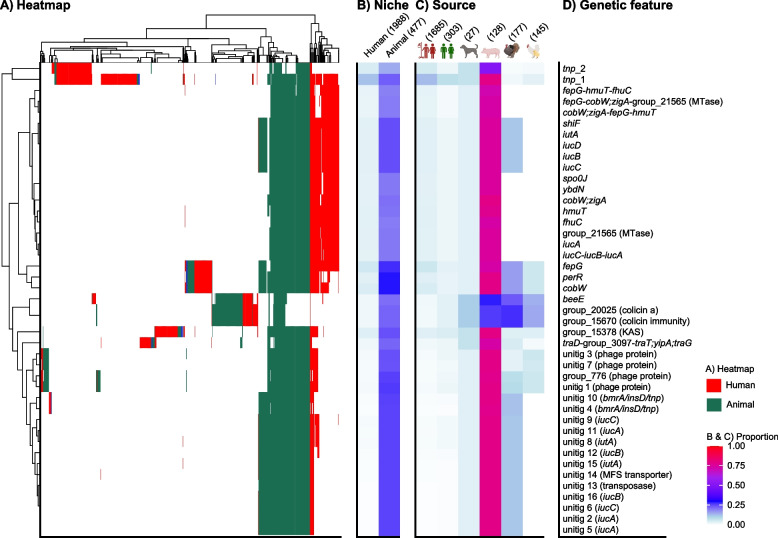
Niche-associated genetic features identified through genome-wide association studies (GWAS).** A** A heatmap showing the presence of significantly associated genetic features (rows), among the 2456 *Klebsiella pneumoniae* genomes from humans and animals (columns), coloured by niche (inset legend). All features were positively associated with the animal niche. **B** Proportions of genomes with genetic features by niche and **C** source. The total number of *K. pneumoniae* genomes per niche and source are indicated on top of the columns. **D** Annotation of genetic features. See Additional file 2: Table S6 and Additional file 3: Fig. S11 for more detail. Genes annotated with more than one name are separated by a semicolon, unitigs found in multiple genes are separated by a slash

We performed GWAS to look for genetic features of the 2,465 K*. pneumoniae* isolates associated with the animal (*n* = 477) or human (*n* = 1988) niches. The marine niche and other KpSC members could not be assessed due to low sample sizes. Overall, 43 genetic features were positively associated with the animal niche and 39/43 (90.7%) were also negatively associated with the human niche: 22 genes (of 36,771 unique among the *K. pneumoniae* genomes), 5 structural genes (of 54,261; i.e., groups of three consecutive genes), 16 unitigs (of 5,688,601), and no SNPs (of 959,726) (Additional file 2: Table S6). These genetic features were enriched in animals, but were also present at low frequencies in some isolates from humans (Fig. 4), indicating that strains, MGEs or plasmids with these features in the human niche may have originated from the animal niche or have become associated with the animal niche once introduced there (we could not infer directionality).

Many of the features were linked to the aerobactin *iuc* locus, which has previously been identified as pig-associated (see above) [17, 18]. Most of the genes and unitigs were co-distributed with *iuc* genes and similarly associated with animals in the GWAS, presumably due to physical linkage on the *iuc*-encoding plasmids (Additional file 3: Fig. S11A), although they were not all exclusively found in *iuc*-encoding genomes (see Additional file 3: Fig. S11B). These features were likely enriched in the animal niche by the increased prevalence of the pig-associated *iuc*3-encoding plasmids. The genes enriched in the animal niche not associated with *iuc* plasmids were two encoding the bacteriocin colicin a and colicin immunity protein (group_20025 and group_15670; see Additional file 2: Table S6), and phage portal protein BeeE (group_15639), which were frequently found together on plasmids, although *beeE* was also found separately on several chromosomes (Additional file 3: Fig. S11B). The colicin genes were found in 27.7% (49/177) of *K. pneumoniae* isolates from turkeys, 26.6% (34/128) pigs, 15.2% (22/145) broilers, and 11.1% (3/27) dogs, compared to 1.6% (27/1685) human infection and 3.6% (11/303) community carriage. There was no clear association with particular clones, except for 15 genomes belonging to SL35 with KL22. Inspection of 14 closed genomes with these features revealed that colicin a, colicin immunity protein and the phage portal protein were co-located on highly similar plasmids (Additional file 3: Fig. S12).

### Lack of niche restriction of KpSC sublineages

Some SLs were broadly distributed across sources: SL17 (*n* = 177), SL37 (*n* = 144), SL45 (*n* = 81), SL200 (*n* = 34), SL29 (*n* = 32), SL34 (*n* = 30) and SL111 (*n* = 21) were identified in all niches (Additional file 2: Table S2). SL107 (*n* = 103), SL35 (*n* = 78), SL641 (*n* = 36) and SL290 (*n* = 35) were found in humans and animals but not marine samples, and SL3010 (*n* = 123), SL10 (*n* = 48), SL25 (*n* = 45) and SL461 (*n* = 33) in humans and marine samples but not animals. The detection of these SLs across niches cannot be interpreted as direct transmission between niches; however, it argues against niche restriction of SLs. Dated phylogenies showed that these clones have existed for a long time: estimated dates for the most recent common ancestors (MRCAs) of isolates of each SL from Norway ranged from 1313 to 1972, with variable uncertainty (Additional file 3: Fig. S13 and Additional file 2: Table S3). Further, a search of publicly available genomes at Pathogenwatch [48] showed that these SLs are widely distributed over time, geography and sources: 4–58 countries, 10–23 years, and at least one genome per SL was found in animal, food, environmental or wastewater samples (Additional file 2: Table S4).

The most prevalent SLs identified in a single niche were found in humans only: SL307 (*n* = 62), SL14 (*n* = 46), SL1562 (*n* = 28), SL15 (*n* = 26), SL268 (*n* = 25), SL2004 (*n* = 25), SL359 (*n* = 23), SL258 (*n* = 22), SL70 (*n* = 21) and SL23 (*n* = 20). This was not surprising as the sample size for humans was much larger than for other niches. However, there was evidence to support that the prevalence of these SLs in non-human samples is actually lower: if the SLs with > 23 human isolates were present in the other niches at the same prevalence as in humans, we would expect to observe them at least once among the animal and marine samples (assessed using a binomial test with the alternative hypothesis that they are less prevalent, see Additional file 2: Table S7A). Therefore, these SLs may be present in the animal and marine sources, but at significantly lower frequencies than among human isolates.

Most capsule biosynthesis (K) (79/131, 60.3%) and lipopolysaccharide (O) loci (10/12, 83.3%) were observed across niches. However, some loci were enriched: O1/O2v2, O3/O3a, O4 and OL103 were enriched in the human niche, whilst O3b and OL13 were enriched in the animal niche (see Additional file 2: Table S7B). Of K loci, KL2, KL10, KL25, KL102 and KL103 were enriched in the human niche; KL14, KL21, KL22, KL30, KL31, KL55 and KL57 in the non-human niche. Several of these K and O loci were associated with particular strains, e.g. SL107, KL103 and O1/O2v1 were found in 86 genomes that were part of a clonal expansion (see below and Additional file 2: Table S7C). K and O loci were relatively stable within SLs, and most SLs were associated with a single K/O combination (82.6%; 708/857), and the same SLs from different sources shared the same K/O loci (Additional file 3: Fig. S14).

### Cross-niche strain-sharing rare, but of potential impact

**Fig. 5:**
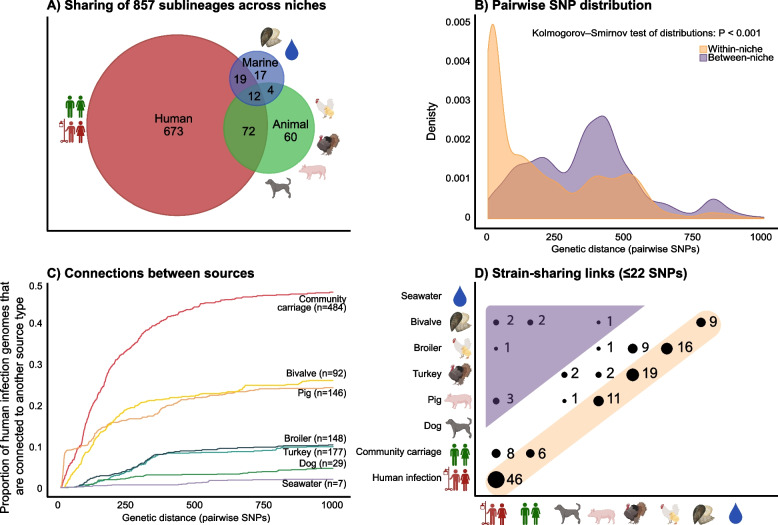
Strain-sharing within and between ecological niches and sources.** A** Venn-diagram showing the distribution of 857 sublineages (SLs) across human, animal and marine niches. In total, 107 SLs were present in ≥ 2 niches. **B** Distribution of pairwise single nucleotide polymorphism (SNP) distances, grouped by genome-pairs within the same niche (orange) and between niches (purple), showing distances up to 1000 SNPs. **C** Connections between sources. The proportion of human infection genomes (*y*-axis) that were connected to one of the other sources, by the number of pairwise SNPs (*x*-axis). **D** Strain-sharing links (clusters of genomes sharing ≤ 22 SNPs) between source pairs, as indicated on the *x*- and *y*-axes. The yellow shading highlights within-source clusters, and the purple highlights the cross-niche clusters

To assess the relatedness of strains within and between niches, we enumerated pairwise SNPs within the 107 niche-overlapping SLs (Fig. 5A). Isolates from the same niche were more closely related (*P* < 0.001, see Fig. 5B), consistent with within-niche transmission. However, we also observed closely related pairs of isolates from different niches. The SNP distances between isolates from human infections and those from other sources showed that human infection isolates shared more recent ancestry with isolates from human community carriage, followed by pigs and bivalves (Fig. 5C). At ≤ 22 SNPs (selected using cutpointR as the optimal threshold for defining strain-sharing genome pairs in this study [see Methods], and in line with thresholds identified in hospital-based studies to identify transmission clusters [5, 49, 50]), 1.9% (41/2172) of human infection isolates were linked to community carriage isolates in our sample (which were restricted to individuals living in the Tromsø municipality, isolated 2015–2016, compared with clinical isolates which were sampled across all Norwegian clinical microbiology laboratories during 2001–2018). At the same genetic distance (≤ 22 SNPs), 4.0% (86/2172) of human infection isolates were linked to pig isolates (*n* = 5; collected in 2019 from 5 farms across the country) and 0.8% (18/2172) to bivalves (*n* = 3; collected in 2016 and 2019 from different locations, two from food production sites).

Using the ≤ 22 SNP threshold, there were 130 within-niche sharing events (60 within the human niche, 61 animal and 9 marine), compared to only nine between the niches (Fig. 5D). Isolate pairs with ≤ 22 SNPs were collected within short time periods (mean 0.7 years, range 0–11). The nine cases of cross-niche strain-sharing were between humans and pigs (1 SL37, 2 SL107), humans and broilers (1 SL200), humans and bivalves (2 SL25, 1 SL461, 1 SL3010), and pigs and bivalves (1 SL3676) (Additional file 3: Fig. S15). Dated phylogenies of SL25, SL107 and SL3010 showed that the cross-niche pairs had MRCAs < 3.8, < 5.5 and < 14.5 years ago, respectively (Additional file 3: Fig. S13).

## Discussion

KpSC infections in humans can be caused by strains circulating within the human population or introduced from animals or the environment as a result of contaminated food or direct contact. Using population genomics in a One Health context, we have shown that the KpSC populations isolated from human, animal and marine sources from Norway are distinct but overlapping. In particular, pigs and marine bivalves appear as potential reservoirs for strains and plasmids that interact with human KpSC populations, by contributing to colonisation and infection in humans, or by being contaminated by human sources via direct or indirect transmission chains. Our study was conducted in a low AMR prevalence context. In the Norwegian surveillance programme for AMR in 2023, only 6.6% of human clinical KpSC isolates were resistant to third-generation cephalosporins and 0.4% to carbapenems [26, 51]. This provided an opportunity to investigate the interactions of KpSC populations without the interference of strong selective pressures from antibiotics.

In total, we identified nine cases of recent strain-sharing between the niches. This national collection spanned a 20-year period, but the samples in the three niches were not matched by year or location. We therefore did not expect to capture direct transmission links. However, the detection of genetically related isolates implies shared ancestry, and thus movement of bacterial strains between niches, although we could not ascertain directionality nor confirm whether movement occurred directly between the sampled niches, via intermediate niches or from a common source. Contemporaneous sampling in a localised area would likely capture more sharing events, however, given the ubiquity and diversity of KpSC within sources it would be difficult to capture evidence of transfer without sampling directly linked sources (e.g. humans and the specific food sources they were recently exposed to).

We found that spillover of strains and clinically relevant genes was more common between sources within the same niche. In the human niche, we found an overlap between carriage and infection. This was not unexpected, as it has been shown that as many as half of hospital-acquired KpSC infections are caused by a patient’s own colonising strain upon admission [5]. There were several strain-sharing events within and between different food-producing animals (pigs, turkeys and broilers). Strain-sharing within the same host may be due to vertical dissemination in animal production pyramids, and sharing both within and between sources may be explained by unsampled transmission from other animals or people, or from e.g. tools, feed or fertiliser [52]. The marine niche was the most underrepresented in our collection (see Table 1). Yet, five of the nine cross-niche events were linked to marine bivalves, and the marine isolates were more similar to the human isolates in terms of heavy metal- and thermoresistance genes compared to the animal isolates. No comparable collections of marine KpSC samples have been published, however, a recent study [53] of the mussel microbiota along the coasts of the Yellow Sea and the East China Sea showed that all samples (*n* = 36) contained species in the Enterobacteriaceae family as well as AMR genes typically harboured by these. They noted that the species (alpha) diversity varied by sample location, likely due to biotic and abiotic variables such as water quality, sediment competition and temperature, and that the microbiota richness and composition can affect colonisation resistance. These variables can influence how well pathogens like KpSC can persist or grow in the mussels, indicating that KpSC prevalence in marine environments in other geographical locations may vary based on these factors. Bivalves are filter feeders that can accumulate pathogens from their surrounding environment. Agricultural runoff or sources of human pollutants, such as wastewater effluent, are likely transmission routes from humans to bivalves [54, 55], and they may also feed back to the human niche via food consumption [56].

Individual events of ecological spillover may be very rare, as our data and other recent studies of KpSC suggest [6, 11, 12, 19]. However, it is arguable that none of these studies have sampled sufficiently from within direct-contact chains to measure the incidence of such events. Still, even rare events can have major consequences in the long term. It is clear that at least some strains of KpSC can transmit easily between humans in hospital settings [25, 57]. Therefore, upon a new strain entering the human niche (e.g. via food consumption), it is possible that it may spread, adapt and evolve depending on exposures, e.g. to other microbes by sharing of MGEs, or to selection dynamics including antibiotics. For example, we observed diverse SL107 isolates in pigs (consistent with persistent circulation in pigs over a decade, see Additional file 3: Fig. S13), as well as a rapid clonal expansion of SL107 in humans (86 cases in 1.6 years, with < 43 pairwise SNPs [median 13], MRCA < 6 years and accounting for 4.5% of the human bloodstream infections). Whilst our data does not tell us about the directionality of movement of SL107 between humans and pigs (the clonal expansion detected in humans was sister to, not emergent from within, the pig clade, see Additional file 3: Fig. S13), it does demonstrate that the same strain can both persist in pigs and spread rapidly in humans. The SL107 strain was largely without AMR or virulence factors, but six human blood isolates had acquired either AMR determinants or virulence factors, likely reflecting shifting selective pressures in the human niche.

There was overall high genetic diversity throughout the dataset, but most prominent in the human niche, which accounted for 43.1% of the unique genes including a wider range of AMR and virulence determinants. This is likely reflective of the larger size of the human niche collection, and that people tend to travel and interact with other people, animals, and environments, thereby facilitating the spread of bacteria. In our previous study of human *K. pneumoniae* carriage, travel was associated with a higher prevalence of carriage [22]. Still, the animal and marine KpSC populations greatly overlapped with those in the human niche. 

Whilst only 2.6% of the genes were unique to the animal niche, we did identify niche-associated traits that were positively associated with animals. They were largely linked with aerobactin-encoding plasmids which were frequent in animals, particularly *iuc*3 in pigs. Aerobactin is a siderophore that increases iron acquisition and can enhance bacterial growth and/or virulence [58]. Aerobactin *iuc*3 is associated with pigs, as previously reported [6, 17, 18, 59], and as confirmed by the GWAS in this study. *iuc*3 is typically located on a conserved transposon on diverse IncFIB(K)-IncFII(K)-plasmids, from many different clonal backgrounds [17, 18]. Although much rarer in clinical cases than the classical KpVP-1/KpVP-2 hypervirulence-associated plasmids encoding *iuc*1 or *iuc*2, KpSC with *iuc*3-encoding plasmids have been reported in invasive human infections [6, 18, 21, 25, 58]. We could not infer directionality, however, the high similarity of plasmids (up to 100% sequence coverage and 0 SNPs) in KpSC isolates from pigs and humans in our study suggests a public health risk potential from animal reservoirs. The mechanisms of adaptation and significance of *iuc*3 in KpSC in pigs remain unclear, and the IncFIB(K)-IncFII(K)-plasmids are largely uncharacterised, but some genes have been described, including genes likely contributing to immune evasion, biofilm formation, arsenic resistance and other genes facilitating iron uptake [17, 18, 58]—many of which we also identified in our GWAS (see Additional file 2: Table S6). Further, the *iuc*3 locus itself may through iron sequestering promote survival in the pig gut by outcompeting other microorganisms in its shared environment. There were also animal-associated genes linked to colicins and phages, which we hypothesise may reflect niche adaptation as a result of interactions with specific bacteria or phages unique to the animal gut microbiomes. Bacteriocins like colicin are peptides that can kill or inhibit the growth of competing species, offering an advantage to the colicin-producing bacteria for surviving in environments with limited nutrients or space [60]. The colicin immunity protein, which was co-located with colicin on the same plasmids, encodes proteins that prevent self-destruction by neutralising the effects of its own colicin, as well as protecting against similar toxins from neighbouring cells [60]. The role of the phage portal protein BeeE in niche adaptation remains unclear. While its presence may suggest adaptation of some form through horizontal gene transfer, all closed plasmid instances of BeeE were co-located with the colicin and colicin immunity proteins (see Additional file 3: Fig. S11B), which could mean that it acts as a passenger rather than a driver of adaptation.

In our collection of 857 SLs, 107 were present in at least two niches. SL17, SL35, SL37, SL45, SL107 and SL3010 were the most common niche-overlapping SLs. SL107 was inflated due to a clonal expansion among human infection isolates [25], whereas SL17, SL37 and SL45 appeared more generalist, being common in all eight source types (except seawater for SL45). SL17 has previously been described as a generalist clone with a wide range of AMR and virulence determinants that can colonise and cause opportunistic infections in both humans and animals [43]. Based on our data, similar conclusions may be drawn for SL35, SL37 and SL45, which were present across the niches, with varying levels of both AMR and virulence. These SLs have also been reported in animals and food products in other studies from Germany, Italy, Thailand, Ghana, Australia and the USA [6, 19, 59, 61–63]. SL107 may also be a generalist clone, it has been observed encoding AMR and/or *iuc*3 in animals, food products and humans in the Netherlands, Germany, Norway, Italy and China [7, 17, 25, 59, 64, 65]. While some clones were more generalist, there were also potential specialist clones within our dataset: SL14, SL15, SL23, SL70, SL147, SL258 and SL307 were highly prevalent and only present in the human niche. These have all been described previously as MDR- or hypervirulence-associated clones that disproportionately contribute to the global burden of hospital-associated disease and nosocomial outbreaks [1].

Another important aspect of One Health is the transfer of MGEs between strains and across niches [66]. As this is a major topic, it will be addressed in a separate study. Here, we focused on the mobility of known clinically relevant features, including virulence and resistance to third-generation cephalosporins and carbapenems. The majority of AMR spread occurred within the human KpSC population. Genes encoding ESBLs or carbapenemases were nearly exclusively in the human niche, except for two ESBL-encoding plasmids identified in a dog and a marine bivalve, which were not related to other strains or plasmids in our dataset. We observed a correlation between the presence of AMR, heavy metal- and thermoresistance genes with specific plasmid replicon markers in the human niche and found the same feature combinations without AMR present in all niches. Further data and analysis are needed to understand if these are the same or similar plasmids with/without the AMR encoding genes. A previous study hypothesised that exposure to antibiotics is the major driver behind co-occurrence of AMR and heavy metal resistance determinants in bacteria from humans and domestic animals [67]. We detected a lower prevalence of these determinants among animal isolates, particularly of plasmid-encoded arsenic, copper and silver-resistance genes, which may play a role in limiting innate immune responses [68, 69]. In contrast, a recent Portuguese study observed that > 60% of poultry KpSC strains from several farms were MDR and resistant to copper and silver with genetic similarities to isolates from human infections [70], showing the potential for animals to be reservoirs for these features if they are exposed. We observed increased levels of cobalt/nickel resistance (*rcnAR)* in animal and marine KpSC compared to human KpSC, which might indicate environmental influences on its distribution. This was the only heavy metal resistance determinant with higher prevalence in animals than humans. *rcnAR* is an efflux system for nickel and cobalt [71], and its presence could be linked to metal exposure in soil or water, although this association has not been described. Further research incorporating environmental data would be needed to clarify the selective pressures shaping its distribution across niches.

Known virulence-encoding genes were most frequent in the animal niche, but consisted of only two lineages: (1) a clonal expansion in turkeys with an *iuc*5 +/− *iro*5-encoding plasmid [23], and (2) the pig-associated aerobactin lineage *iuc*3, which we observed in pigs [17], a dog, human infections and community carriage. Global epidemiological analyses of the *iuc*3 lineage previously revealed that most of the *iuc*3-encoding plasmids recovered from humans in our study were related to plasmids originating from Asia or Europe, but two of the plasmids were likely the result of spillover of strains between pigs and humans in Norway [17, 18]. Several reports have shown the emergence of convergent KpSC strains carrying both AMR genes and hypervirulence traits [1, 72]. We observed only six such cases, but the recent findings of plasmids harbouring both *iuc*3 and ESBLs in Thailand in both humans and animals [18] calls for concern given the high prevalence of *iuc*3 in our dataset.

Our overall findings are similar to a KpSC One Health study from Italy and confirm on a much broader scale those from the UK, the USA, the Caribbean, Ghana and India [6, 11, 12, 19, 20, 73]. These studies suggested that infection prevention measures should be focused on clinical settings to limit the spread of MDR and to break nosocomial transmission chains. While we agree, we also emphasise that a One Health surveillance framework remains important. KpSC classically causes opportunistic nosocomial infections, however, hypervirulent and sporadic classic infections also occur in healthy individuals in the community, which cannot be prevented by hospital infection control measures [1, 25]. Therefore, other interventions that prevent or limit transmission and subsequent colonisation from the food chain or direct contact with animals are also important.

Ecological barriers such as geographical distance or physical barriers are likely limiting exposure (opportunity for frequent strain transmission) between niches more than the genetic make-up of the KpSC themselves. We have here shown that approximately half the genes in the pangenome overlap the three ecological niches, and a recent study also found considerable overlap of accessory genes between the community carriage isolates in Norway and isolates from Ghana [19]. One Health studies of KpSC to date have found limited overlap of AMR between humans and animals [6, 11, 12, 19, 20, 73], however, whilst the prevalence may be low, MDR and/or ESBL KpSC strains have been found in animals, vegetables and animal food products from several geographical regions, with similarities to clinical strains, including global MDR-associated clones [20, 70, 74–80]. A study performed in six countries in Europe found little overlap of AMR between food items and human-polluted environments [81]. In contrast, a systematic review of 38 studies from low- and middle-income countries showed that food-producing animals are important reservoirs for clinically relevant resistance determinants and urged that increased surveillance is necessary for aiding prevention in a similar manner to that developed by the USA and countries in the European Union [82]. Another study found that AMR-encoding bacteria were distributed among humans, animals and the environment in Tanzania [83], noting that the amount of direct or indirect contact between food-animal reservoirs and people affects the amount of transmission and that this varies based on socio-economic, cultural and ecological contexts. Thus, the low rates of AMR, virulence and strain transmission across niches in our study and in other high-income countries likely reflect the efficiency of existing preventative measures, highlighting the success of One Health initiatives rather than diminishing their importance.

## Conclusions

Our findings, together with One Health studies in other settings, indicate that human-to-human transmission of bacterial strains and AMR is more frequent than between ecological niches, and even more so within clinical settings. However, our study also identified the animal niche as a reservoir for virulent strains that may spill over to humans and cause highly pathogenic infections. Taken together with growing evidence of convergent plasmids also in non-human niches, these observations reinforce the notion that the potential for public health implications from even rare zoonotic transmission must not be underestimated. Our findings support the case for surveillance of bacteria in a One Health perspective even in a low AMR setting like Norway, as it enables the detection of emergence and spread of resistant or pathogenic strains that could have significant public health impacts. Increased population densities and environmental changes will likely lead to more frequent interactions within and between human, animal and environmental niches, which will facilitate zoonotic transmission and the formation of new reservoirs, underscoring the need for One Health surveillance approaches globally.

## Supplementary Information

Additional file 1. Supplementary methods.

Additional file 2. Supplementary tables. Table S1: Genotyping results and QC. Table S2: Source-overlap by sublineage. Table S3: BactDating results. Table S4: Genomes included in BactDating analyses. Table S5: Chi-squared tests of AMR presence by niche. Table S6: GWAS results. Table S7: Binomial tests of SLs, KLs and OLs by human vs non-human sources. Table S8: Genome accessions and metadata.

Additional file 3. Supplementary figures. Fig. S1. Strain-sharing pairs by single nucleotide polymorphism (SNP) thresholds. Fig. S2. QQ-plots of the pyseer results. Fig. S3. Antimicrobial resistance (AMR) genes and mutations by source. Fig. S4. Comparison of clinically relevant features across niches. Fig. S5. Recognised multidrug resistance (MDR)- and hypervirulence-associated clones. Fig. S6. Virulence factors by source. Fig. S7. Distribution of heavy metal- and thermoresistance genes by replicon type. Fig. S8. Distribution of heavy metal- and thermoresistance operons/genes by source. Fig. S9. Plasmid replicon markers associated with clinically relevant features. Fig. S10. Co-occurrence of genetic features within genomes. Fig. S11. Presence of niche-associated genetic features by niche and replicon type. Fig. S12. Distribution of colicin genes. Fig. S13. Dated trees of prevalent niche-overlapping SLs. Fig. S14. Capsule (K) and O loci by source and sublineages (SLs). Fig. S15. Strain-sharing clusters by source.

## Data Availability

All Illumina and Oxford Nanopore Technologies reads are available under the umbrella project PRJEB74192 on the European Nucleotide Archive. The hybrid-assembled genomes have been deposited in GenBank. See Additional file 2: Table S8 for read and biosample accessions and metadata, and Additional file 2: Table S1 for the genotyping results. The code used for analyses and to create figures, as well as the nucleotide sequences of the significant GWAS genes and unitigs, are available in: Hetland MAK, et al., https://github.com/marithetland/KGWP1_crossniche; 10.5281/zenodo.15112620 (2025).

## Abbreviations

AMR: Antimicrobial resistance
CG: Clonal group
cgMLST: Core genome multilocus sequence type
ESBL: Extended-spectrum β-lactamase
GWAS: Genome-wide association studies
KpSC: *Klebsiella pneumoniae* species complex
LIN: Life identification numbers
MDR: Multidrug-resistant
MGE: Mobile genetic element
MRCA: Most recent common ancestor
SL: Sublineage
SNP: Single nucleotide polymorphism
ST: Sequence type
