# Supplementary material for: A genome-wide One Health study of Klebsiella pneumoniae in Norway reveals overlapping populations but few recent transmission events across reservoirs
Source: Genome Med. 2025 Apr 28;17:42. doi: 10.1186/s13073-025-01466-0 (PMC12039103; doi:10.1186/s13073-025-01466-0)
Supplement: Supplementary file 1 — Additional file 1. Supplementary methods. [file 13073_2025_1466_MOESM1_ESM.pdf]

# Supplementary methods

## A genome-wide One Health study of *Klebsiella pneumoniae* in Norway reveals overlapping populations but few recent transmission events across reservoirs

Marit A K Hetland [1,2,a], Mia A Winkler [1,3], Håkon P Kaspersen [4], Fredrik Håkonsholm [3,5], Ragna-Johanne Bakksjø [1], Eva Bernhoff [1], Jose F. Delgado-Blas [6], Sylvain Brisse [6], Annapaula Correia [7], Aasmund Fostervold [1,8], Margaret M C Lam [9], Bjørn-Tore Lunestad [2,5], Nachiket P Marathe [5], Niclas Raffelsberger [3,10], Ørjan Samuelsen [11], Marianne Sunde [12], Arnfinn Sundsfjord [3,11], Anne Margrete Urdahl [4], Ryan R Wick [13], Iren H Löhr [1,8,b], Kathryn E Holt [7,9,b]

**Affiliations:**

[1] Department of Medical Microbiology, Stavanger University Hospital, Stavanger, Norway
[2] Department of Biological Sciences, Faculty of Science and Technology, University of Bergen, Bergen, Norway
[3] Department of Medical Biology, Faculty of Health Sciences, UiT The Arctic University of Norway, Tromsø, Norway
[4] Research Section Food Safety and Animal Health, Department of Animal Health and Food Safety, Norwegian Veterinary Institute, Ås, Norway
[5] Institute of Marine Research, Bergen, Norway
[6] Biodiversity and Epidemiology of Bacterial Pathogens Unit, Institut Pasteur, Université Paris Cité, Paris, France
[7] Department of Infection Biology, Faculty of Infectious and Tropical Diseases, London School of Hygiene & Tropical Medicine, London, United Kingdom
[8] Department of Clinical Science, Faculty of Medicine, University of Bergen, Bergen, Norway
[9] Department of Infectious Diseases, School of Translational Medicine, Monash University, Melbourne, Australia
[10] Department of Microbiology and Infection Control, University Hospital of North Norway, Tromsø, Norway
[11] Norwegian National Advisory Unit on Detection of Antimicrobial Resistance, Department of Microbiology and Infection Control, University Hospital of North Norway, Tromsø, Norway
[12] Section for Bacteriology, Department for Analysis and Diagnostics, Norwegian Veterinary Institute, Ås, Norway
[13] Department of Microbiology and Immunology, University of Melbourne at the Peter Doherty Institute for Infection and Immunity, Melbourne, Australia

[a] Corresponding author: Marit A. K. Hetland, marit.andrea.klokkhammer.hetland@sus.no
[b] These authors contributed equally

**Keywords**: One Health, *Klebsiella pneumoniae* species complex, genomics, GWAS, AMR, transmission, zoonotic transmission, ecology

**Selection of isolates for long-read sequencing**

All 3,255 *Klebsiella pneumoniae* species complex (KpSC) isolates were short-read sequenced as previously described [1-9]. We also included long-read Oxford Nanopore Technology (ONT) sequences of 16.9% (n=550/3255) of the isolates to produce hybrid assemblies. Of the 550 isolates, 27 were selected and sequenced as part of previously published studies [1,6,8,9], 359 were selected to span the genetic diversity of the dataset (based on the pangenome matrix of the short-read data, using the long-read selection tool [10], downloaded on 2022-01-27) and to include at least one genome per sublineage (SL) that overlapped the three niches (n=107 SLs). We also included isolates based on plasmid replicon markers (n=104) and virulence or AMR determinants (n=60) in the short-read assemblies.

**Quality control**

To ensure the quality of the genome data included in this study, we used the following tools to inspect the quality of the Illumina and ONT reads: fast_count (https://github.com/rrwick/MinION-desktop/blob/master/fast_count.cpp) to inspect the number of reads, average read lengths and read depth (number of bases divided by the total assembly length); Kleborate v2.4.0 [11] to inspect the species match, contig count, total assembly length, N50, largest contig, and ambiguous bases; Quast v5.2.0 [12] to inspect GC%; and KmerFinder v3.0.2 [13] to inspect contamination levels (i.e. the top matching species and the percentage of hits that did not match that species). The quality control results are available in Table S1.

**Identification of heavy metal operons and thermoresistance genes**

We searched the annotated assemblies for experimentally confirmed heavy metal resistance genes listed in the Antibacterial Biocide and Metal Resistance Genes (BacMet) database (http://bacmet.biomedicine.gu.se/index.html) [14]. Heavy metal resistance is typically encoded by operons of genes, where certain genes must be present for the operon to be active. This has been experimentally confirmed for some metals in some bacterial species, but far from all. Based on literature searches, we used the following operons to determine presence or absence of heavy metal resistance: Arsenic resistance was determined by the presence of at least *arsABCDR* [15-17]. Resistance to chromium was defined by the presence of *chrA*. The plasmid-encoded *chrB1* is believed to increase resistance but is not essential [15,18]. Mercury resistance was determined by the presence of at least *merAPR* and either *merC, merF, or merT* [15,19]. Copper resistance operons were defined as *cusABCF*, *pcoABCDRS* or *copABCD* [15,20]. Resistance to nickel was defined as the presence of *ncrABC* [21]. Resistance to cadmium was defined as *cadABC* [17,22,23]. Silver resistance was defined as the presence of at minimum *silABCERS* [15]. Several operons were used to determine tellurite resistance, including *klaABC*, *kilA* and *telAB*, *tehAB,* and *terBCDE* [24]. The presence of *zitB* genes indicated resistance to zinc, and the *zntAR* operon defined resistance to cadmium, lead, and zinc [25,26]. The *czcCBA* operon was used to detect the multi-metal efflux pump for cadmium, zinc, and cobalt [15,22]. The presence of *rcnAR* defined resistance to cobalt and nickel [27]. There were no complete operons of *klaABC, kilA, telAB, czcCBA,* or *cusABCF* in any genomes in the overall collection and they were therefore not shown in figures/data. The following operons were present

in ≥99% of genomes in all niches and were therefore excluded from the figures: *cadABC* (n=3,244), *cueOR* (n=3,249), *tehAB* (n=3,254), *zitB* (n=3,253), and *zntAR* (n=3,245). Thermoresistance was defined as the presence of the genes *clpK* or *hsp20* [28,29]. We utilised our hybrid genome assembly collection (n=550/3,255) to determine if these genes/loci were most commonly encoded on plasmids or on chromosomes (Fig. S7).

**Inferring cross-talk between niches**

Of the 107 niche-overlapping SLs, we selected those that were represented by ≥20 genomes and had been collected over at least a 5-year period (n=15 SLs). We first estimated dated phylogenies using both our local dataset and publicly available genomes for robust clock rate estimates. We then estimated dated phylogenies of the local datasets only, using the best fit clock models from the global phylogenies, and setting the mutation rates identified from the global phylogenies as the initial rate of substitutions per genome.

For each SL, we downloaded all publicly available genomes (short-reads) with known year of collection identified on https://pathogen.watch on 01.07.2023. They were assembled with the same methods as the local genomes (TrimGalore v0.6.7 [https://github.com/FelixKrueger/TrimGalore] and SPAdes v3.15.4 [30]). This gave a total number of 2,974 public and 1,020 local genomes for these analyses. For each SLs, both the public and local genomes were included to identify and filter recombinations using verticall v0.4.2 (https://github.com/rrwick/Verticall). The output was used by BactDate v1.1.1 [31] to infer dated phylogenies of each SL. We specified exact dates of sampling for genomes where this was available (n=707 from our local dataset), and for genomes where only sampling year was available we specified a year range (n=313 from our dataset, all of the public genomes) (Table S4 lists the accessions and relevant metadata).

BactDating was run with three clock models, a strict (“strictgamma”), mixed (“mixedgamma”) and relaxed log normal clock model (“relaxedgamma”), with a constant coalescent demographic model. Each model was run in three independent replicates, to $10^8$ Markov Chain Monte Carlo (MCMC) iterations, until all parameters reached >200 effective sampling size (ESS). For each SL, the best fit model was determined using the modelcompare function in BactDate.

Prior to running BactDate, we tested for temporal signal (association between time and genetic divergence) using the root-to-tip regression function in BactDate with the verticall tree. The root-to-tip genetic distances were positively associated with the dates of isolation (Table S3), indicating molecular clock signals. After running BactDate, the statistical significance of the temporal signal was tested by running the same model again but with sampling dates set equal, and then comparing the two models.

To assess the relatedness of respectively colicin-containing and *iuc*3-containing plasmids across the niches, RedDog v1beta.11 (https://github.com/katholt/RedDog) was used to align all 3,255 short-read genomes against the largest closed colicin-containing (KGVET-2020-01-4249-1, 54.9 kbp) and *iuc*3-containing (KGVETS-2019-01-1806, 148.4 kbp) plasmids from the collection and comparing the number of single nucleotide polymorphisms (SNPs) and replicon coverage. To compare the structure of the

colicin-containing genomes, Clinker v0.0.29 [32] was used to align all closed colicin-containing plasmid sequences.

### Assessing niche enrichment

To assess niche-enrichment, we performed genome-wide association studies (GWAS) with pyseer v1.3.11 [33]. We looked for associations using four genetic features: 1) the gene presence absence matrix from panaroo v1.3.3 [34], 2) the structural presence absence matrix from panaroo, 3) SNPs generated with RedDog mapping against the reference SGH10 (GenBank accession no CP025080.1, re-annotated here with Bakta v1.8.1 [35]), and 4) unitigs generated with unitig-caller v1.3.0 (https://github.com/bacpop/unitig-caller). The analysis was restricted to *K. pneumoniae* due to differences in KpSC species distribution and counts in the three niches.

We used the linear mixed model (LMM) in pyseer, accounting for population structure by using a maximum likelihood tree generated by IQ-tree v2.2.6 [36] from the same alignment as we derived the SNPs from above. The pyseer-script 'phylogeny_distance.py' was used to midpoint root the tree and to calculate the distance matrix needed for population structure correction. For pyseer, we set the minimum allele frequency to 5% and the maximum to 95%, to exclude very rare variants and variants that were very common in the dataset from the analysis. QQ-plots of the pyseer results revealed that the population structure had not been sufficiently accounted for. We therefore manually decreased the p-value thresholds for significant associations based on the QQ-plots (Fig. S2). Genes, structural genes and SNPs were identified based on the Bakta-annotations. Unitig locations were identified by searching the Bakta-annotated hybrid genome assembly collection (n=550/3,255).

### References

(1) Fostervold A, Hetland MAK, Bakksjø R, et al. A nationwide genomic study of clinical Klebsiella pneumoniae in Norway 2001-15: introduction and spread of ESBLs facilitated by clonal groups CG15 and CG307. *J Antimicrob Chemother*. 2022;77(3):665-674. doi:10.1093/jac/dkab463

(2) Fostervold A, Raffelsberger N, Hetland MAK, et al. Risk of death in Klebsiella pneumoniae bloodstream infections is associated with specific phylogenetic lineages. *J Infect*. 2024;88(5):106155. doi:10.1016/j.jinf.2024.106155

(3) Raffelsberger N, Hetland MAK, Svendsen K, et al. Gastrointestinal carriage of Klebsiella pneumoniae in a general adult population: a cross-sectional study of risk factors and bacterial genomic diversity. *Gut Microbes*. 2021;13(1):1939599. doi:10.1080/19490976.2021.1939599

(4) Håkonsholm F, Hetland MAK, Svanevik CS, Sundsfjord A, Lunestad BT, Marathe NP. Antibiotic Sensitivity Screening of *Klebsiella* spp. and *Raoultella* spp. Isolated from Marine Bivalve Molluscs Reveal Presence of CTX-M-Producing *K. pneumoniae*. *Microorganisms*. 2020;8(12):1909. doi:10.3390/microorganisms8121909

(5) Håkonsholm F, Hetland MAK, Svanevik CS, Lunestad BT, Löhr IH, Marathe NP. Insights into the genetic diversity, antibiotic resistance and pathogenic potential of Klebsiella pneumoniae from the Norwegian marine environment using whole-genome analysis. *Int J Hyg Environ Health*. 2022;242:113967. doi:10.1016/j.ijheh.2022.113967

(6) Håkonsholm F, Hetland MAK, Löhr IH, Lunestad BT, Marathe NP. Co-localization of clinically relevant antibiotic- and heavy metal resistance genes on plasmids in Klebsiella pneumoniae from marine bivalves. *Microbiologyopen*. 2023;12(4):e1368. doi:10.1002/mbo3.1368
(7) Franklin-Alming FV, Kaspersen H, Hetland MAK, et al. Exploring *Klebsiella pneumoniae* in Healthy Poultry Reveals High Genetic Diversity, Good Biofilm-Forming Abilities and Higher Prevalence in Turkeys Than Broilers. *Front Microbiol*. 2021;12:725414. doi:10.3389/fmicb.2021.725414
(8) Kaspersen H, Urdahl AM, Franklin-Alming FV, et al. Population dynamics and characteristics of *Klebsiella pneumoniae* from healthy poultry in Norway. *Front Microbiol*. 2023;14:1193274. doi:10.3389/fmicb.2023.1193274
(9) Kaspersen H, Franklin-Alming FV, Hetland MAK, et al. Highly conserved composite transposon harbouring aerobactin *iuc3* in *Klebsiella pneumoniae* from pigs. *Microb Genom*. 2023;9(2):mgen000960. doi:10.1099/mgen.0.000960
(10) Arredondo-Alonso S, Pöntinen AK, Cléon F, et al. A high-throughput multiplexing and selection strategy to complete bacterial genomes. *Gigascience*. 2021;10(12):giab079. doi:10.1093/gigascience/giab079
(11) Lam MMC, Wick RR, Watts SC, Cerdeira LT, Wyres KL, Holt KE. A genomic surveillance framework and genotyping tool for *Klebsiella pneumoniae* and its related species complex. *Nat Commun*. 2021;12(1):4188. doi:10.1038/s41467-021-24448-3
(12) Gurevich A, Saveliev V, Vyahhi N, Tesler G. QUAST: quality assessment tool for genome assemblies. *Bioinformatics*. 2013;29(8):1072-1075. doi:10.1093/bioinformatics/btt086
(13) Hasman H, Saputra D, Sicheritz-Ponten T, et al. Rapid whole-genome sequencing for detection and characterization of microorganisms directly from clinical samples. *J Clin Microbiol*. 2014;52(1):139-146. doi:10.1128/JCM.02452-13
(14) Pal C, Bengtsson-Palme J, Rensing C, Kristiansson E, Larsson DG. BacMet: antibacterial biocide and metal resistance genes database. *Nucleic Acids Res*. 2014;42(Database issue):D737-D743. doi:10.1093/nar/gkt1252
(15) Silver S, Phung le T. A bacterial view of the periodic table: genes and proteins for toxic inorganic ions. *J Ind Microbiol Biotechnol*. 2005;32(11-12):587-605. doi:10.1007/s10295-005-0019-6
(16) Mukhopadhyay R, Rosen BP, Phung LT, Silver S. Microbial arsenic: from geocycles to genes and enzymes. *FEMS Microbiol Rev*. 2002;26(3):311-325. doi:10.1111/j.1574-6976.2002.tb00617.x
(17) González Henao S, Ghneim-Herrera T. Heavy metals in soils and the remediation potential of bacteria associated with the plant microbiome. *Front Environ Sci*. 2021;9. doi: 10.3389/fenvs.2021.604216
(18) Cervantes C, Campos-García J, Devars S, et al. Interactions of chromium with microorganisms and plants. *FEMS Microbiol Rev*. 2001;25(3):335-347. doi:10.1111/j.1574-6976.2001.tb00581.x
(19) Barkay T, Miller SM, Summers AO. Bacterial mercury resistance from atoms to ecosystems. *FEMS Microbiol Rev*. 2003;27(2-3):355-384. doi:10.1016/S0168-6445(03)00046-9
(20) Rensing C, Grass G. Escherichia coli mechanisms of copper homeostasis in a changing environment. *FEMS Microbiol Rev*. 2003;27(2-3):197-213. doi:10.1016/S0168-6445(03)00049-4
(21) Hufnagel DA, Choby JE, Hao S, et al. Antibiotic-Selected Gene Amplification Heightens Metal Resistance. *mBio*. 2021;12(1):e02994-20. doi:10.1128/mBio.02994-20
(22) Nies DH. Resistance to cadmium, cobalt, zinc, and nickel in microbes. *Plasmid*. 1992;27(1):17-28. doi:10.1016/0147-619x(92)90003-s
(23) Lee SW, Glickmann E, Cooksey DA. Chromosomal locus for cadmium resistance in Pseudomonas putida consisting of a cadmium-transporting ATPase and a MerR family response regulator. *Appl Environ Microbiol*. 2001;67(4):1437-1444. doi:10.1128/AEM.67.4.1437-1444.2001
(24) Chasteen TG, Fuentes DE, Tantaleán JC, Vásquez CC. Tellurite: history, oxidative stress, and molecular mechanisms of resistance. *FEMS Microbiol Rev*. 2009;33(4):820-832. doi:10.1111/j.1574-6976.2009.00177.x

(25) Maunders EA, Ganio K, Hayes AJ, et al. The Role of ZntA in Klebsiella pneumoniae Zinc Homeostasis. *Microbiol Spectr*. 2022;10(1):e0177321. doi:10.1128/spectrum.01773-21
(26) Grass G, Fan B, Rosen BP, Franke S, Nies DH, Rensing C. ZitB (YbgR), a member of the cation diffusion facilitator family, is an additional zinc transporter in Escherichia coli. *J Bacteriol*. 2001;183(15):4664-4667. doi:10.1128/JB.183.15.4664-4667.2001
(27) Rodrigue A, Effantin G, Mandrand-Berthelot MA. Identification of rcnA (yohM), a nickel and cobalt resistance gene in Escherichia coli. *J Bacteriol*. 2005;187(8):2912-2916. doi:10.1128/JB.187.8.2912-2916.2005
(28) Boll EJ, Frimodt-Møller J, Olesen B, Krogfelt KA, Struve C. Heat resistance in extended-spectrum beta-lactamase-producing Escherichia coli may favor environmental survival in a hospital setting. *Res Microbiol*. 2016;167(5):345-349. doi:10.1016/j.resmic.2016.02.002
(29) Bojer MS, Struve C, Ingmer H, Hansen DS, Krogfelt KA. Heat resistance mediated by a new plasmid encoded Clp ATPase, ClpK, as a possible novel mechanism for nosocomial persistence of Klebsiella pneumoniae. *PLoS One*. 2010;5(11):e15467. doi:10.1371/journal.pone.0015467
(30) Bankevich A, Nurk S, Antipov D, et al. SPAdes: a new genome assembly algorithm and its applications to single-cell sequencing. *J Comput Biol*. 2012;19(5):455-477. doi:10.1089/cmb.2012.0021
(31) Didelot X, Croucher NJ, Bentley SD, Harris SR, Wilson DJ. Bayesian inference of ancestral dates on bacterial phylogenetic trees. *Nucleic Acids Res*. 2018;46(22):e134. doi:10.1093/nar/gky783
(32) Gilchrist CLM, Chooi YH. clinker & clustermap.js: automatic generation of gene cluster comparison figures. *Bioinformatics*. 2021;37(16):2473-2475. doi:10.1093/bioinformatics/btab007
(33) Lees JA, Galardini M, Bentley SD, Weiser JN, Corander J. pyseer: a comprehensive tool for microbial pangenome-wide association studies. *Bioinformatics*. 2018;34(24):4310-4312. doi:10.1093/bioinformatics/bty539
(34) Tonkin-Hill G, MacAlasdair N, Ruis C, et al. Producing polished prokaryotic pangenomes with the Panaroo pipeline. *Genome Biol*. 2020;21(1):180. doi:10.1186/s13059-020-02090-4
(35) Schwengers O, Jelonek L, Dieckmann MA, Beyvers S, Blom J, Goesmann A. Bakta: rapid and standardized annotation of bacterial genomes via alignment-free sequence identification. *Microb Genom*. 2021;7(11):000685. doi:10.1099/mgen.0.000685
(36) Minh BQ, Schmidt HA, Chernomor O, et al. IQ-TREE 2: New Models and Efficient Methods for Phylogenetic Inference in the Genomic Era. *Mol Biol Evol*. 2020;37(5):1530-1534. doi:10.1093/molbev/msaa015
