# Supplementary material for: A genome-wide One Health study of Klebsiella pneumoniae in Norway reveals overlapping populations but few recent transmission events across reservoirs
Source: Genome Med. 2025 Apr 28;17:42. doi: 10.1186/s13073-025-01466-0 (PMC12039103; doi:10.1186/s13073-025-01466-0)
Supplement: Supplementary file 3 — Additional file 3. Supplementary figures. Fig. S1. Strain-sharing pairs by single nucleotide polymorphism (SNP) thresholds. Fig. S2. QQ-plots of the pyseer results. Fig. S3. Antimicrobial resistance (AMR) genes and mutations by source. Fig. S4. Comparison of clinically relevant features across niches. Fig. S5. Recognised multidrug resistance (MDR)- and hypervirulence-associated clones. Fig. S6. Virulence factors by source. Fig. S7. Distribution of heavy metal- and thermoresistance genes by replicon type. Fig. S8. Distribution of heavy metal- and thermoresistance operons/genes by source. Fig. S9. Plasmid replicon markers associated with clinically relevant features. Fig. S10. Co-occurrence of genetic features within genomes. Fig. S11. Presence of niche-associated genetic features by niche and replicon type. Fig. S12. Distribution of colicin genes. Fig. S13. Dated trees of prevalent niche-overlapping SLs. Fig. S14. Capsule (K) and O loci by source and sublineages (SLs). Fig. S15. Strain-sharing clusters by source. [file 13073_2025_1466_MOESM3_ESM.pdf]

Supplementary figures

A genome-wide One Health study of *Klebsiella pneumoniae* in Norway reveals overlapping populations but few recent transmission events across reservoirs

Marit A K Hetland [1,2,a], Mia A Winkler [1,3], Håkon P Kaspersen [4], Fredrik Håkonsholm [3,5], Ragna-Johanne Bakksjø [1], Eva Bernhoff [1], Jose F. Delgado-Blas [6], Sylvain Brisse [6], Annapaula Correia [7], Aasmund Fostervold [1,8], Margaret M C Lam [9], Bjørn-Tore Lunestad [2,5], Nachiket P Marathe [5], Niclas Raffelsberger [3,10], Ørjan Samuelsen [11], Marianne Sunde [12], Arnfinn Sundsfjord [3,11], Anne Margrete Urdahl [4], Ryan R Wick [13], Iren H Löhr [1,8,b], Kathryn E Holt [7,9,b]

Affiliations:

[1] Department of Medical Microbiology, Stavanger University Hospital, Stavanger, Norway

[2] Department of Biological Sciences, Faculty of Science and Technology, University of Bergen, Bergen, Norway

[3] Department of Medical Biology, Faculty of Health Sciences, UiT The Arctic University of Norway, Tromsø, Norway

[4] Research Section Food Safety and Animal Health, Department of Animal Health and Food Safety, Norwegian Veterinary Institute, Ås, Norway

[5] Institute of Marine Research, Bergen, Norway

[6] Biodiversity and Epidemiology of Bacterial Pathogens Unit, Institut Pasteur, Université Paris Cité, Paris, France

[7] Department of Infection Biology, Faculty of Infectious and Tropical Diseases, London School of Hygiene & Tropical Medicine, London, United Kingdom

[8] Department of Clinical Science, Faculty of Medicine, University of Bergen, Bergen, Norway

[9] Department of Infectious Diseases, School of Translational Medicine, Monash University, Melbourne, Australia

[10] Department of Microbiology and Infection Control, University Hospital of North Norway, Tromsø, Norway

[11] Norwegian National Advisory Unit on Detection of Antimicrobial Resistance, Department of Microbiology and Infection Control, University Hospital of North Norway, Tromsø, Norway

[12] Section for Bacteriology, Department for Analysis and Diagnostics, Norwegian Veterinary Institute, Ås, Norway

[13] Department of Microbiology and Immunology, University of Melbourne at the Peter Doherty Institute for Infection and Immunity, Melbourne, Australia

[a] Corresponding author: Marit A. K. Hetland, marit.andrea.klokkhammer.hetland@sus.no

[b] These authors contributed equally

Keywords: One Health, *Klebsiella pneumoniae* species complex, genomics, GWAS, AMR, transmission, zoonotic transmission, ecology

Contents

Fig. S1. Strain-sharing pairs by single nucleotide polymorphism (SNP) thresholds.
Fig. S2. QQ-plots of the pyseer results.
Fig. S3. Antimicrobial (AMR) genes and mutations by source.
Fig. S4. Comparison of clinically relevant features across niches.
Fig. S5. Recognised multidrug resistance (MDR)- and hypervirulence-associated clones.
Fig. S6. Virulence factors by source.
Fig. S7. Distribution of heavy metal- and thermoresistance genes by replicon type.
Fig. S8. Distribution of heavy metal- and thermoresistance operons/genes by source.
Fig. S9. Plasmid replicon markers associated with clinically relevant features.
Fig. S10. Co-occurrence of genetic features within genomes.
Fig. S11. Presence of niche-associated genetic features by niche and replicon type.
Fig. S12. Distribution of colicin genes.
Fig. S13. Dated trees of prevalent niche-overlapping SLs.
Fig. S14. Capsule (K) and O loci by source and sublineages (SLs).
Fig. S15. Strain-sharing clusters by source.

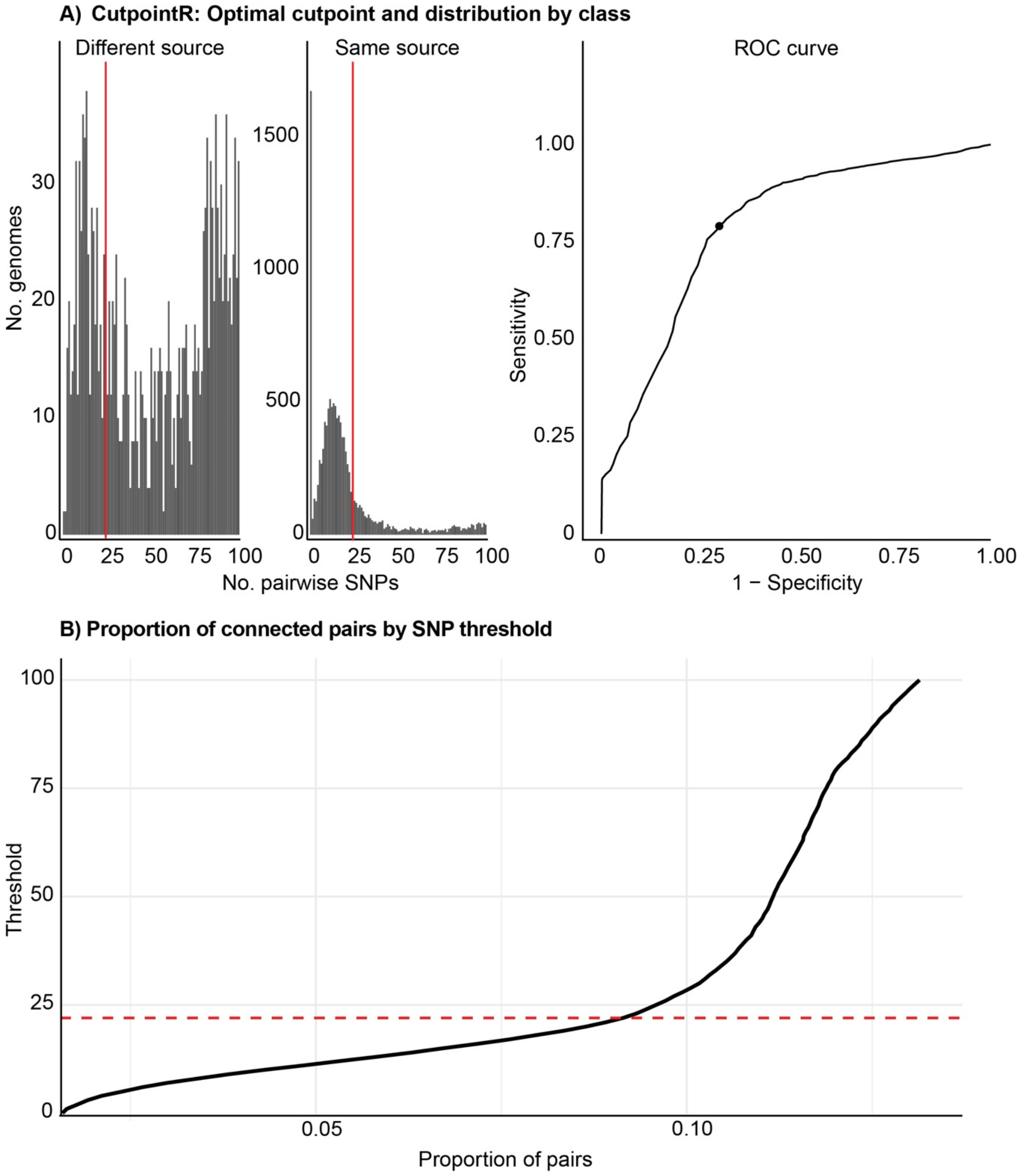

Fig. S1. Strain-sharing pairs by single nucleotide polymorphism (SNP) thresholds. A) CutpointR was used to estimate an optimal SNP threshold of 22 (red line) for defining strain-sharing genome pairs. **B)** The proportion of genome pairs (black line) given a SNP threshold (up to 100 SNPs). The red dotted line shows that the estimated optimal threshold of 22 is at an inflection point in the curve, suggesting it is an appropriate threshold for maximising the differentiation of strain-sharing pairs within or between sources.

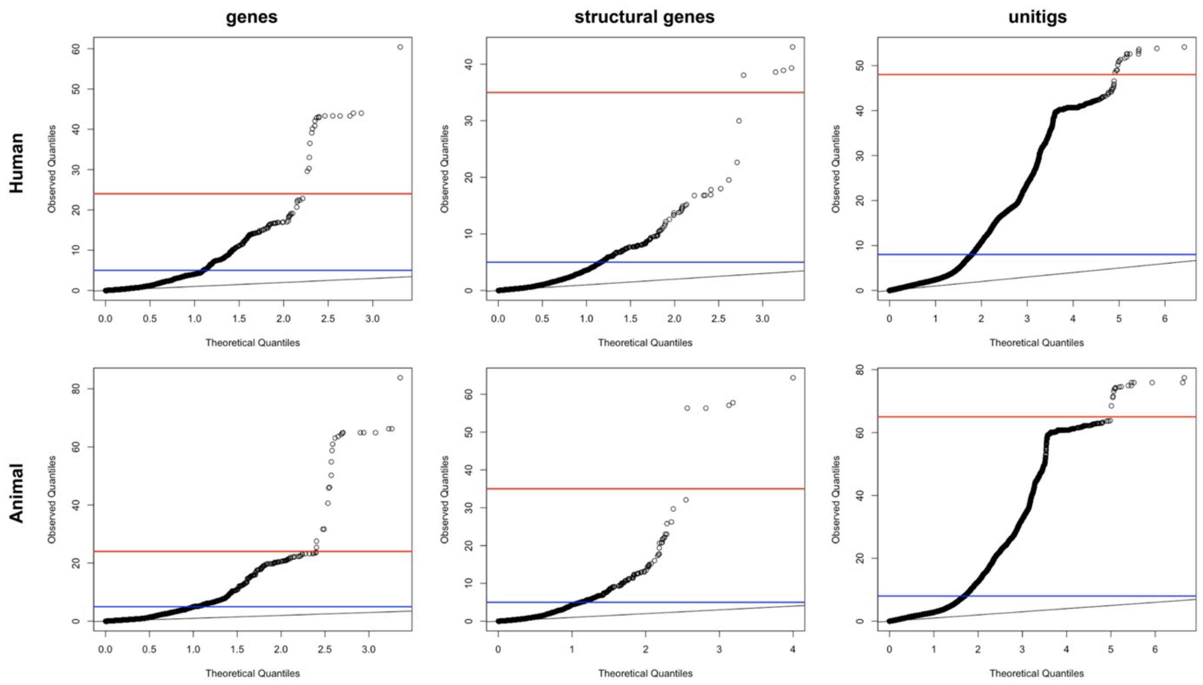

Fig. S2. QQ-plots of the pyseer results. The blue line represents the p-value threshold suggested by pyseer, which was too high because the population structure had not been completely corrected for. The red line indicates a manually set p-value threshold, which was used instead, based on visual inspection of the data.

Class	Determinant	Human infection (2172)	Community carriage (484)	Dog (29)	Pig (146)	Turkey (177)	Broiler (148)	Bivalve (92)	Seawater (7)
Trimethoprim	*dfrA14*	245	7	1				1	
	dfrA12	45	1					1	
	dfrA1	18					1		
Tetracyclines	*tet(D)*	105	10	2		7		3	
	tet(B)	13			1	7			
	tet(A)	211	2			2	5	1	
Sulfonamides	*sul3*	6		1				1	
	sul2	292	8	1	1	6		2	
	sul1	170	5	1			4		
Phenicols	*cmlA1*	7		1				1	
	catA2	21						1	
	catA1	30		1		1			
Fluoroquinolones	ParC-80I	134		1					
	GyrA-83I	108		1					
	qnrS1	53	1		1		1	1	
Fosfomycin	*fosA7*	26	7		1	8	11		
Beta-lactamases w/ inhibitor resistance	SHV-52	7	1		1				
	SHV-187+69L	1					2		
Beta-lactamases	TEM-1D	267	8	2	1	6	1	3	
	LAP-2	25					1		
ESBLs	CTX-M-3	4						1	
	CTX-M-15	205		1					
Aminoglycosides	*strB*	302	12	1	3		1	1	1
	strA	299	10	1	4		1	1	
	aph(3')-Ia	73	5		1		1	1	
	aadA2	61	2	1			3	1	
	aadA	47	3	2				1	
	aac(3)-IId	40		1					

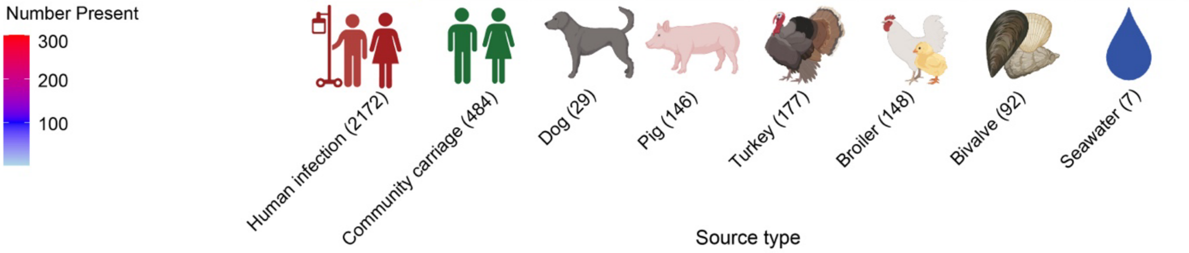

Fig. S3. Antimicrobial resistance (AMR) genes and mutations by source. The number of genomes per source (columns) that have the AMR gene or mutation (determinant) specified in the rows. The AMR determinants are ordered by the AMR class they belong to. In total, 130 AMR determinants spanning 14 AMR classes were observed, of which 127 were found among human infections. Here, only AMR determinants that were also found in ≥1 non-human isolate are shown. The determinants were identified with Kleborate, and includes all complete hits and those with mismatching nucleotides or incomplete (>90%) coverage.

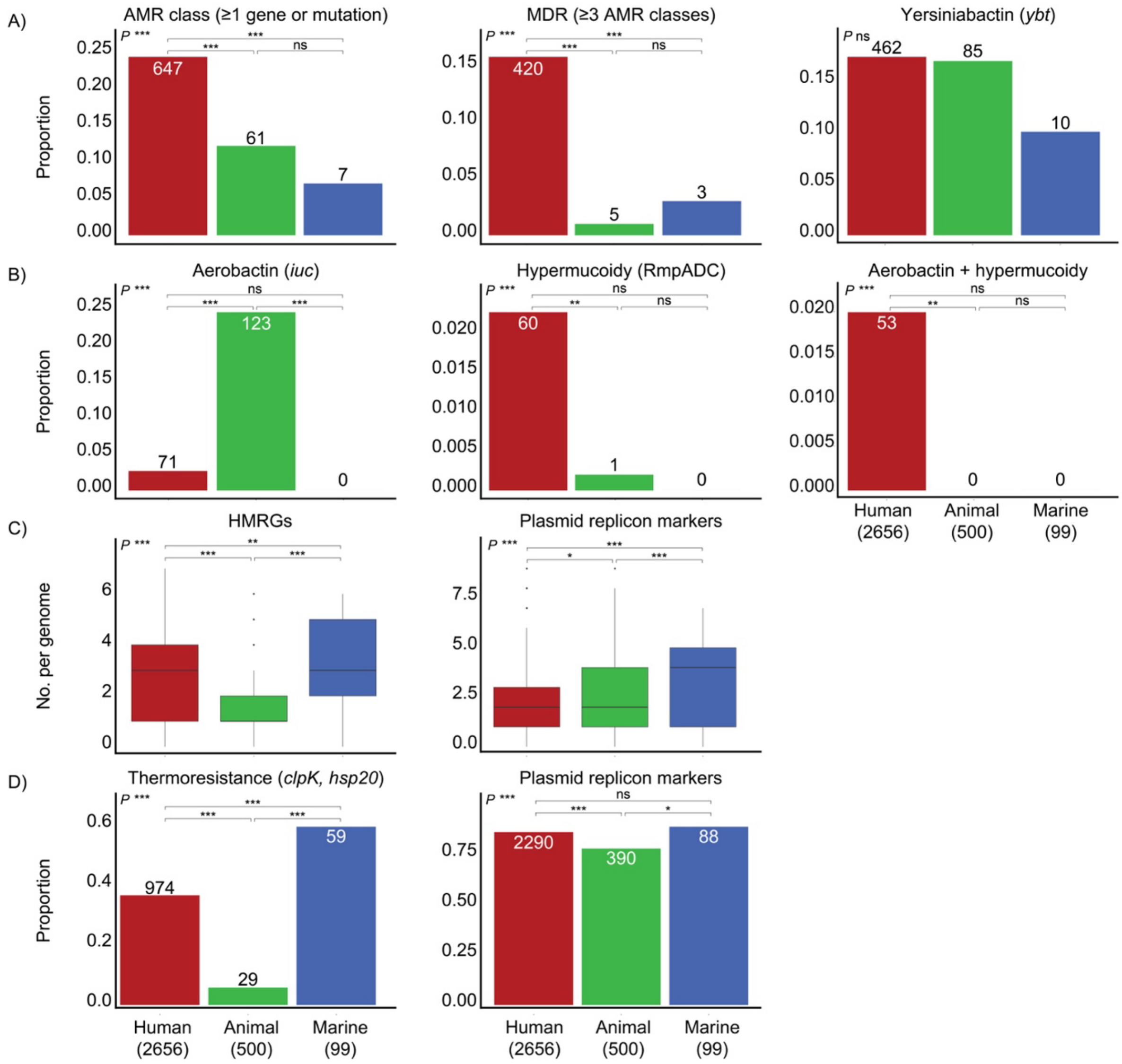

Fig. S4. Comparison of clinically relevant features across niches. A and B) Proportion of antimicrobial resistance (AMR), multidrug resistance (MDR) and virulence factors; siderophores (yersiniabactin and aerobactin), the hypermucoidy locus RmpADC, and combinations of aerobactin and RmpADC, which are indicators of a possible hypervirulent phenotype. The number of genomes per niche is displayed on/in the bars. **C) and D)** Proportion and ranges of heavy-metal resistance operons (HMRGs), plasmid replicon markers and thermoresistance genes. Statistical comparisons were performed using Kruskal-Wallis (overall) and Mann-Whitney (pairwise) tests for ranges, and chi-squared tests for proportions (overall and pairwise). Significance is denoted as follows: * P<0.05, ** P<0.01, *** P<0.001, ns P≥0.05.

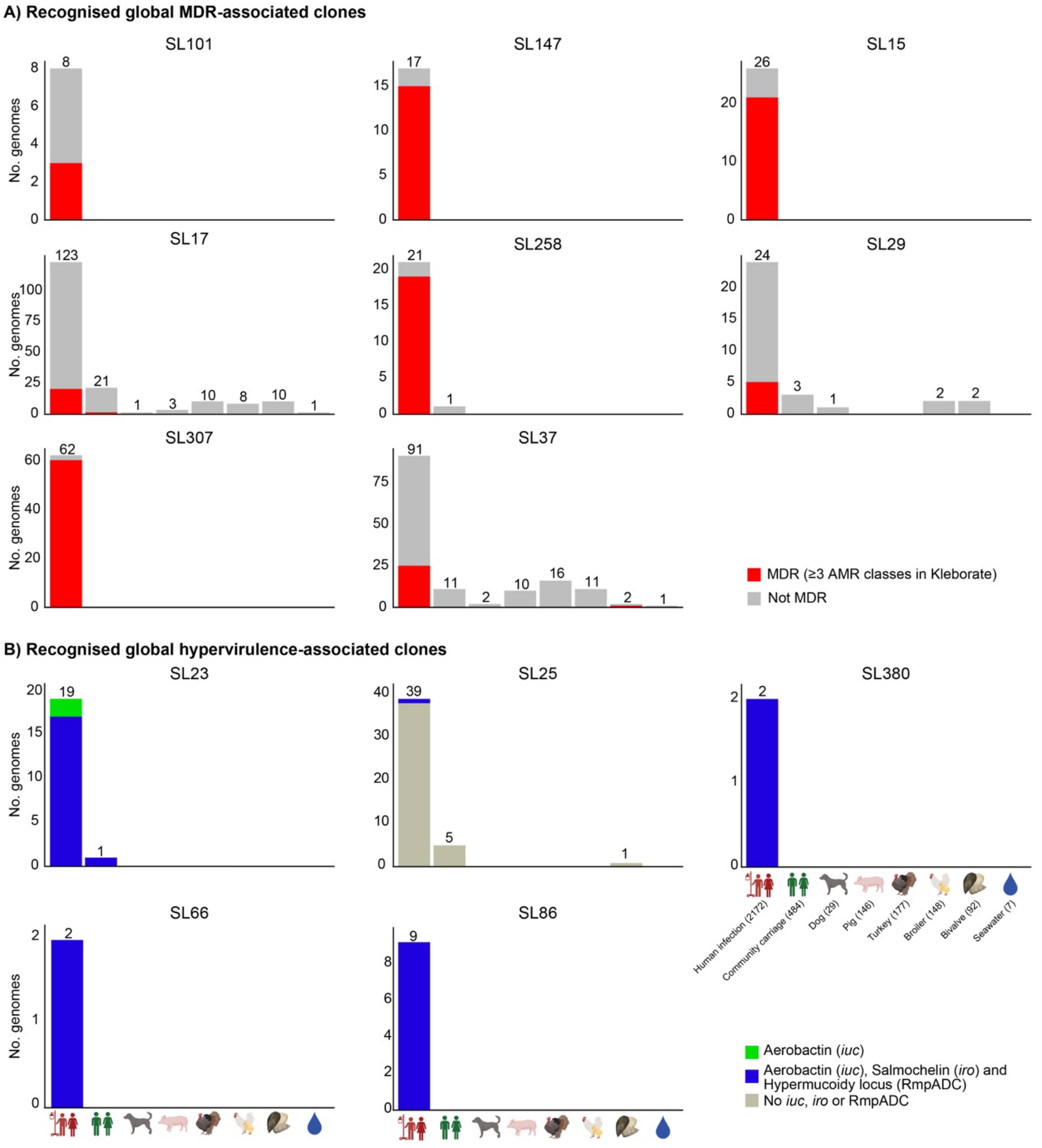

Fig. S5. Recognised multidrug resistance (MDR)- and hypervirulence-associated clones. A) Number of genomes belonging to MDR associated clones (facet), shown by source (x-axis) and coloured by whether they were MDR (red) or not. **B)** Number of genomes belonging to hypervirulence associated clones (facet), shown by source (x-axis) and coloured by virulence loci that were present (inset legend). The total number of genomes per source with these features is indicated on top of the bars.

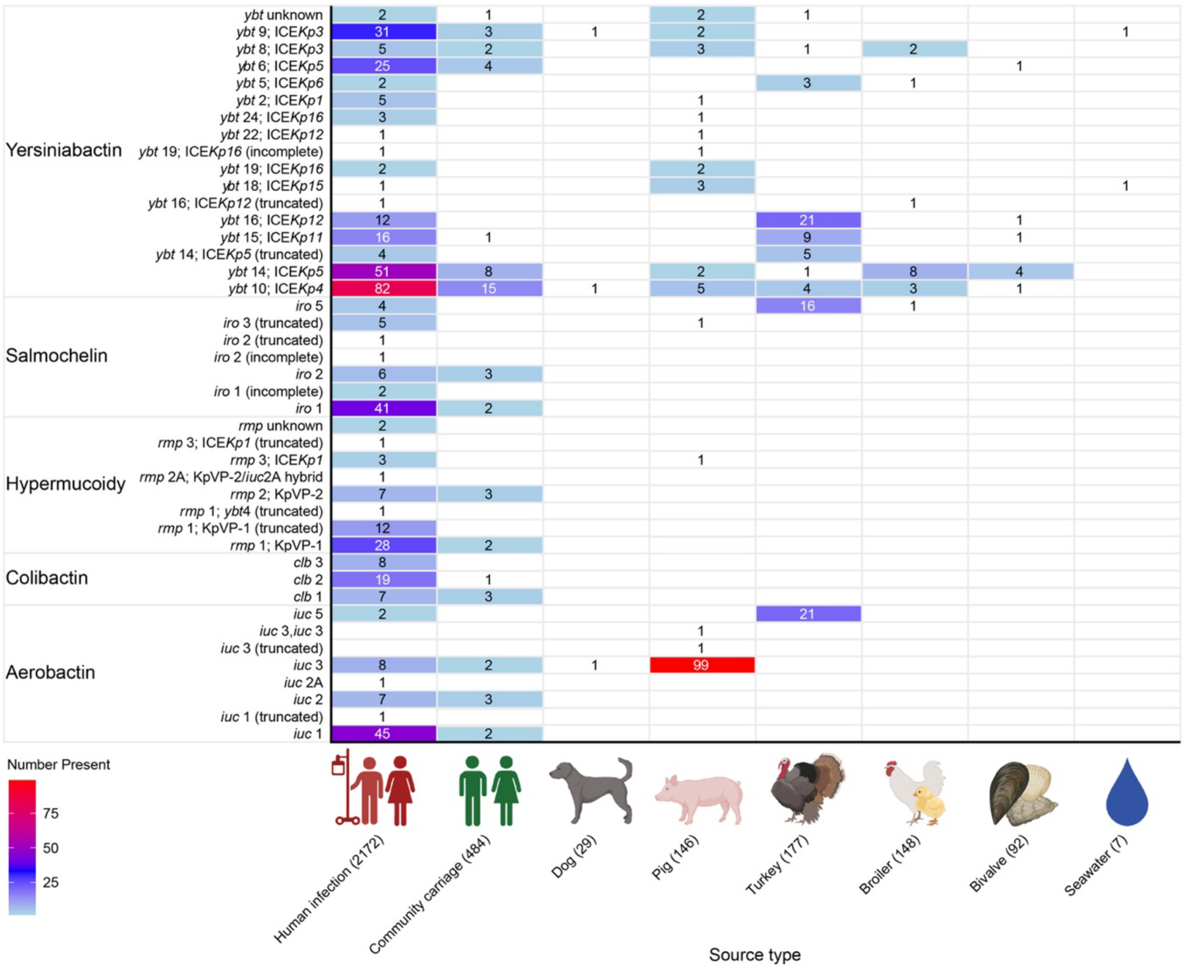

Fig. S6. Virulence factors by source. The number of genomes per source (columns) that have the virulence locus specified in the rows. All loci are shown except for yersiniabactin, where only loci present in ≥1 non-human source are displayed (i.e. 17/63 loci).

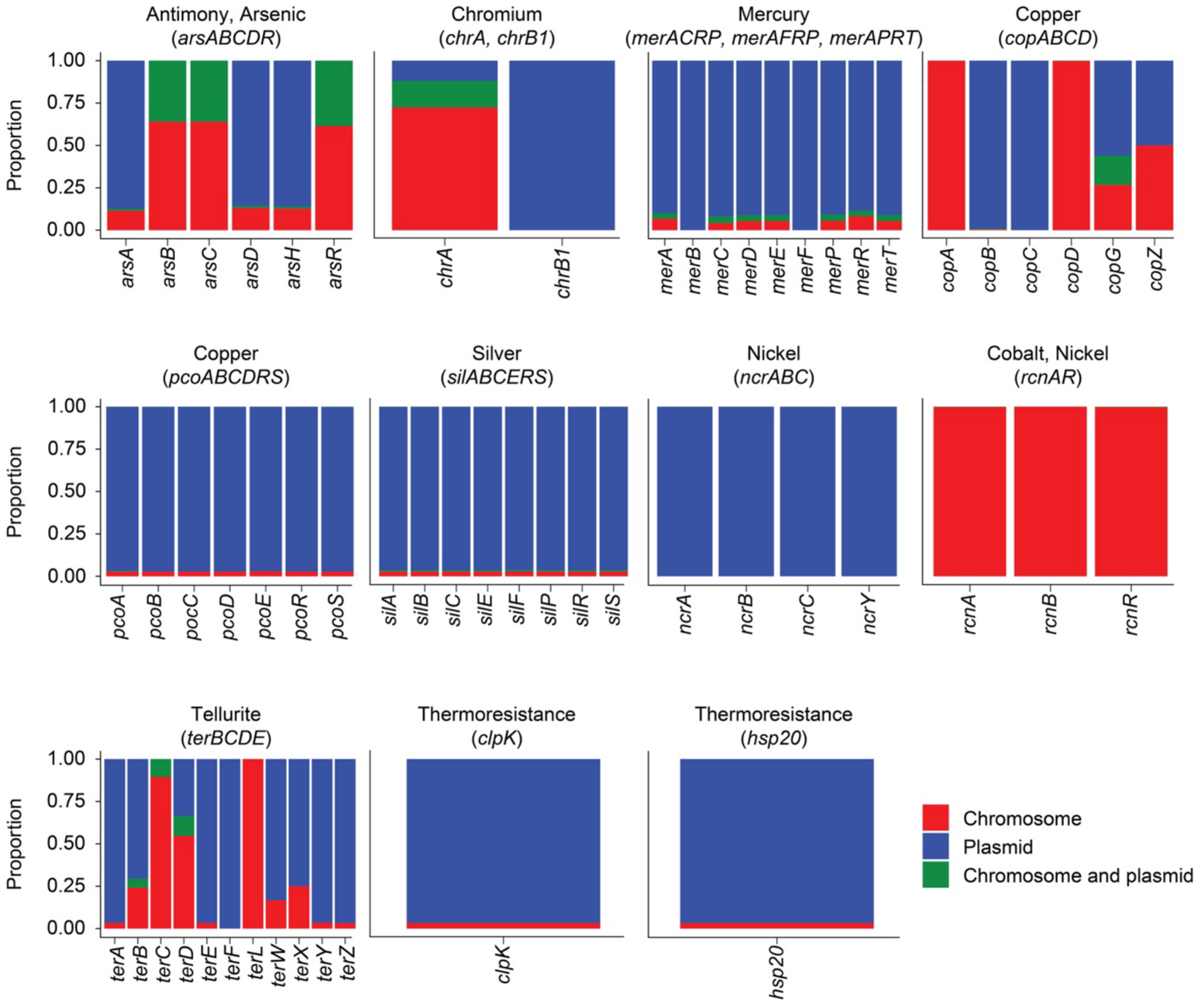

Fig. S7. Distribution of heavy metal- and thermoresistance genes by replicon type. The closed genome collection of 550/3,255 genomes was utilised to determine if features were more commonly present on chromosomes or plasmid sequences. The proportions of genes are coloured by whether the gene was found on the chromosome (red), on plasmids (blue) or on both within the same genome (green). Note that not all genes shown in this figure were essential to the operons, only those in the header brackets (see Supplementary Methods for details).

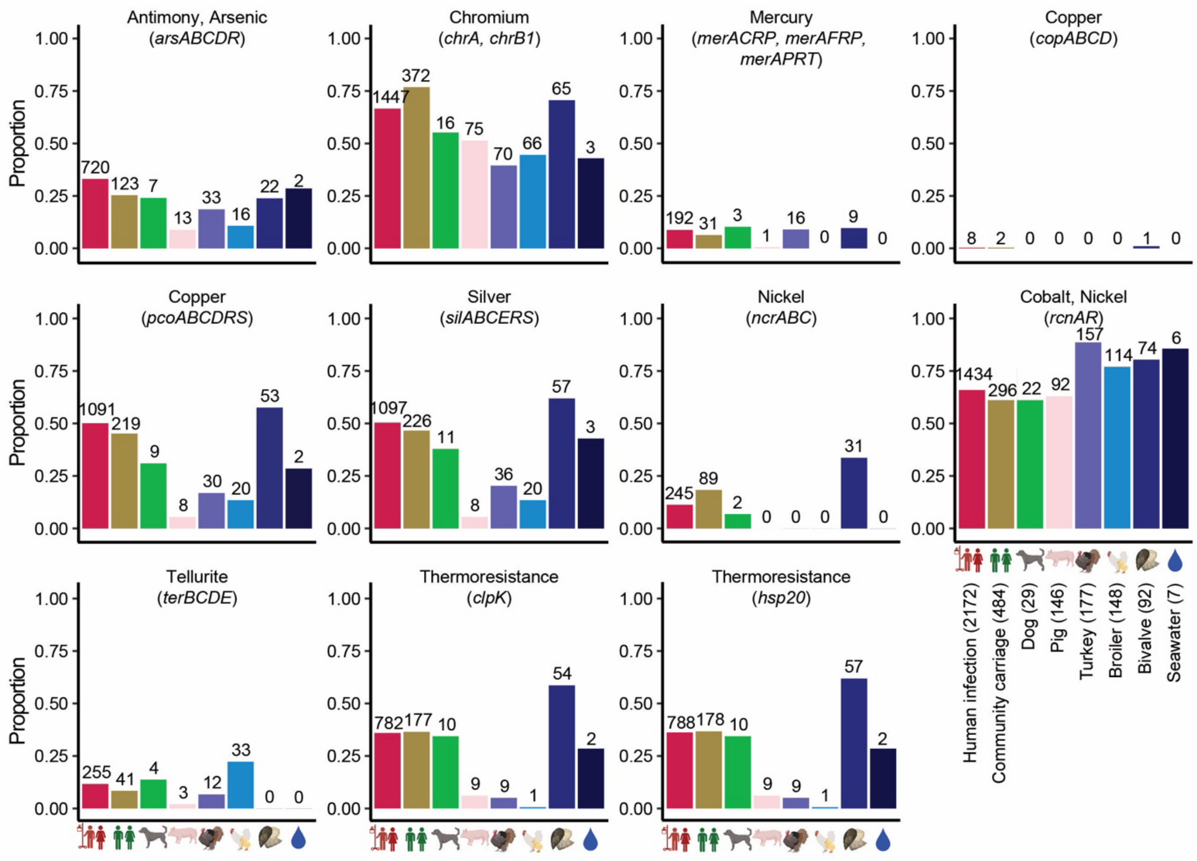

Fig. S8. Distribution of heavy metal- and thermoresistance operons/genes by source. The facets show the presence of different heavy metal or thermoresistance genes or operons by source. The bars show the proportion of genomes by source; the number of genomes is indicated on top of each bar.

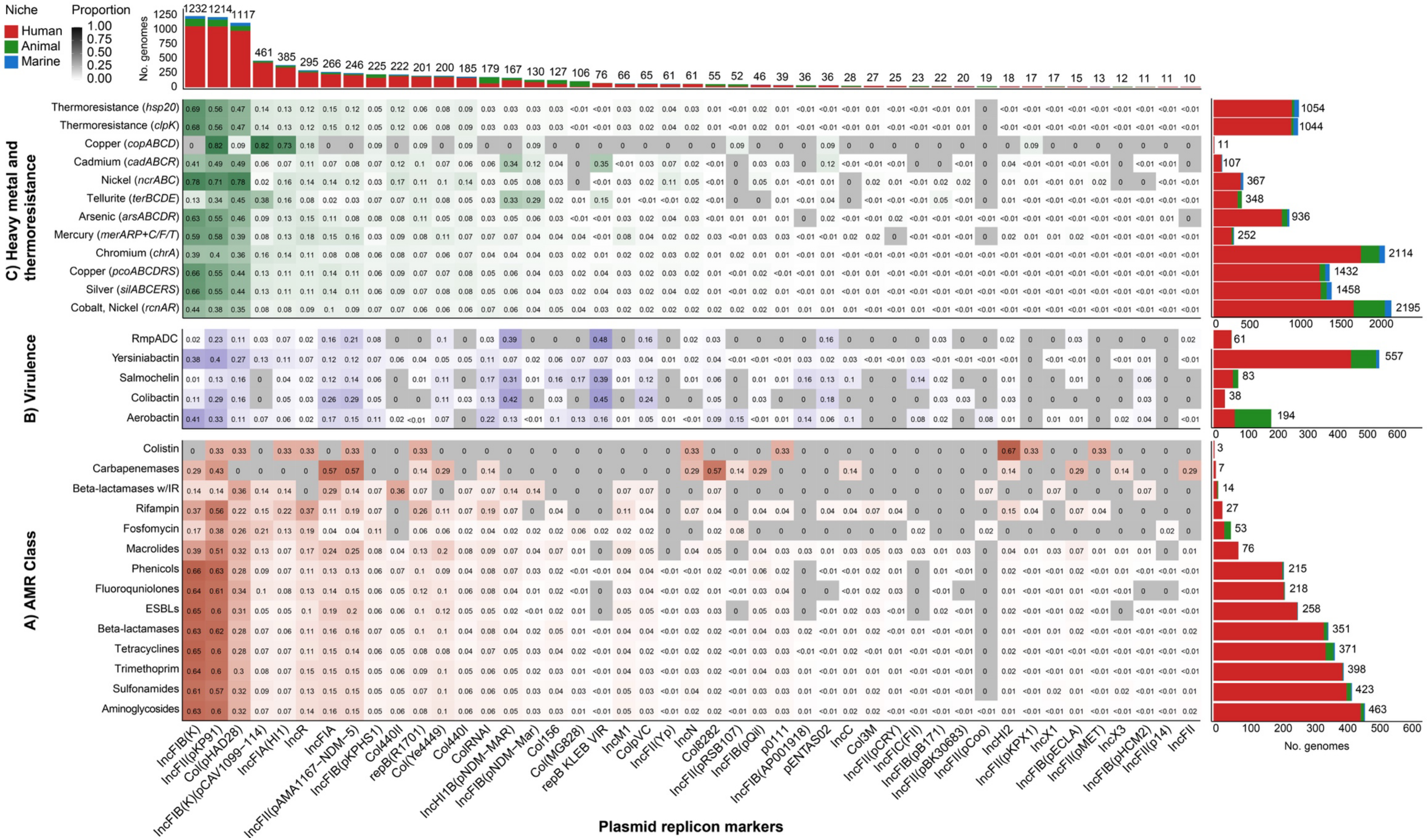

Fig. S9. Plasmid replicon markers associated with clinically relevant features. Proportion of genomes with plasmid replicon markers (x-axis, showing markers found in ≥10 genomes each, i.e. 45 of in total 85 plasmid replicon markers) found together with **A)** Acquired antimicrobial resistance (AMR) determinants by class, **B)** Acquired virulence factors, and **C)** Heavy metal- and thermoresistance operons/genes. The marginal histograms show the number of genomes containing each feature, coloured by niche.

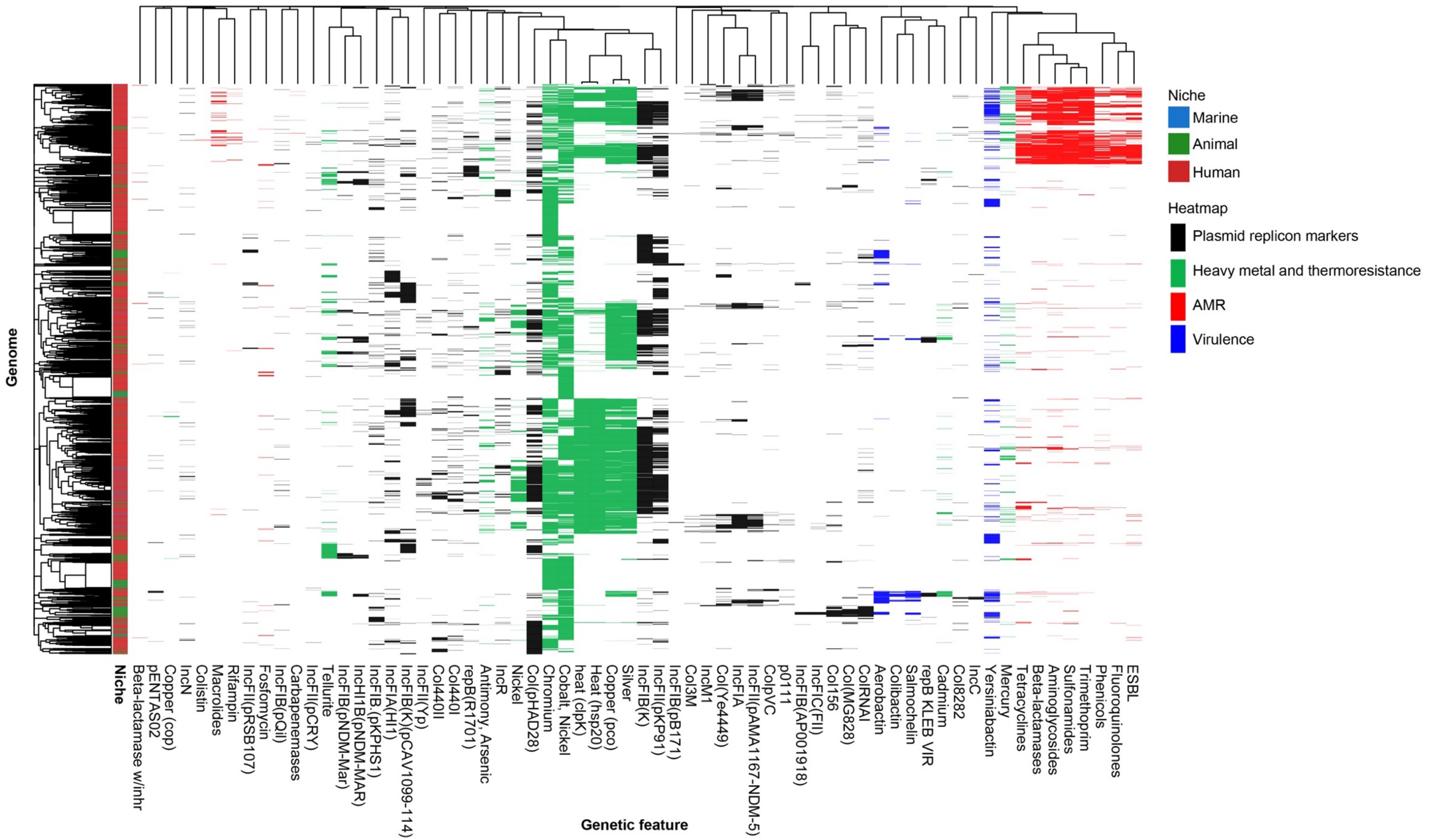

Fig. S10. Co-occurrence of genetic features within genomes. A clustered heatmap showing the presence of genetic features (x-axis) in genomes (y-axis) and the niche they belong to (left-most column, as per inset legend). The features are grouped and coloured by: antimicrobial resistance (AMR) classes, virulence factors, heavy metal- and thermoresistance operons/genes, and plasmid replicon markers that were present in >20 genomes.

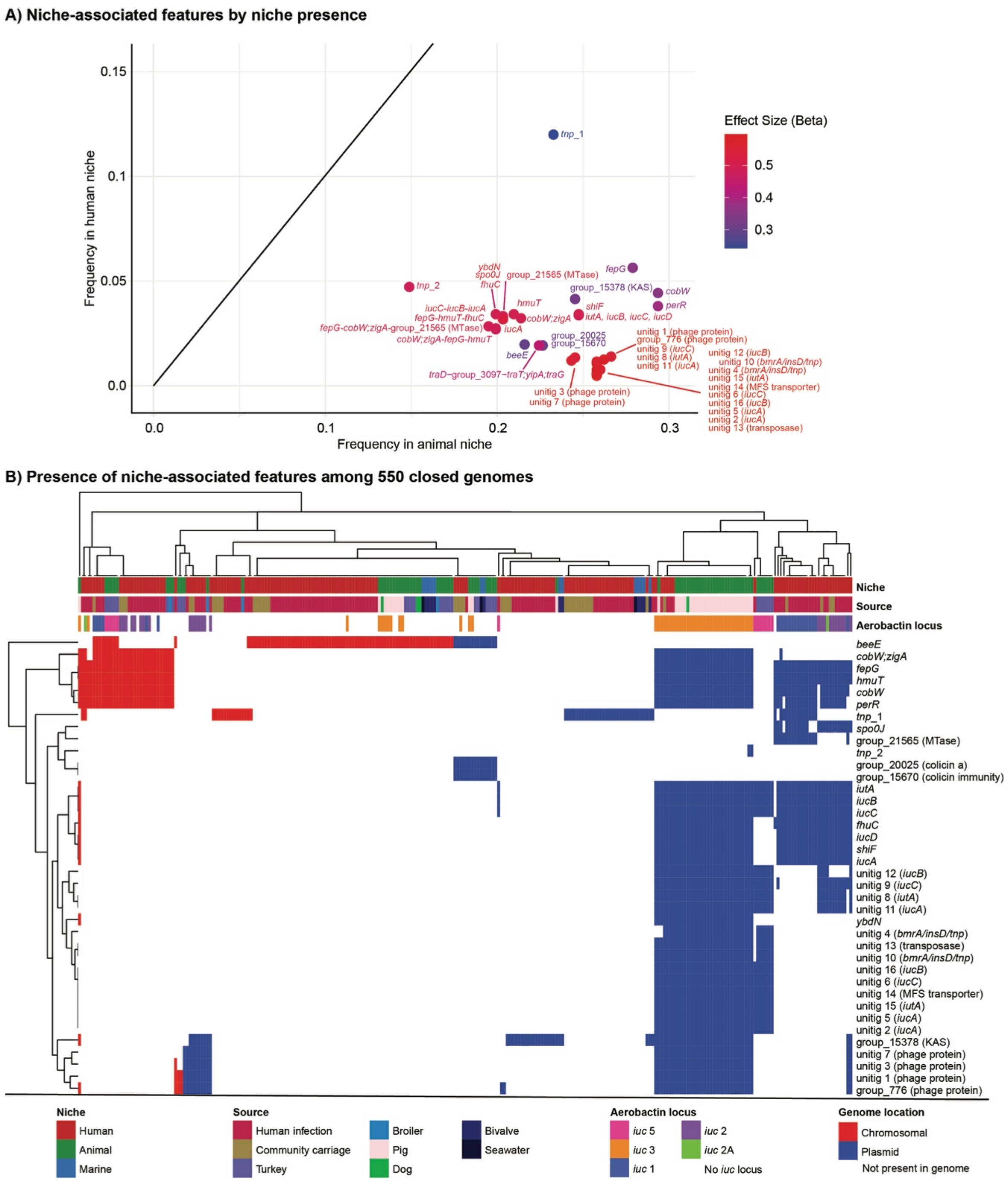

Fig. S11. Presence of niche-associated genetic features by niche and replicon type. A) Niche-associated genetic features by their proportional presence in isolates with the phenotype (x-axis, i.e. in *Klebsiella pneumoniae* isolates from animals) or without (y-axis, i.e. in *K. pneumoniae* isolate from humans). **B)** Presence of significant features in the closed genome collection (n=550/3,255). The heatmap shows if the hits were found on chromosomes (red) or plasmids (blue). The three columns above the heatmap show niche, source and the presence of aerobactin (*iuc*) locus per genome (inset legend).

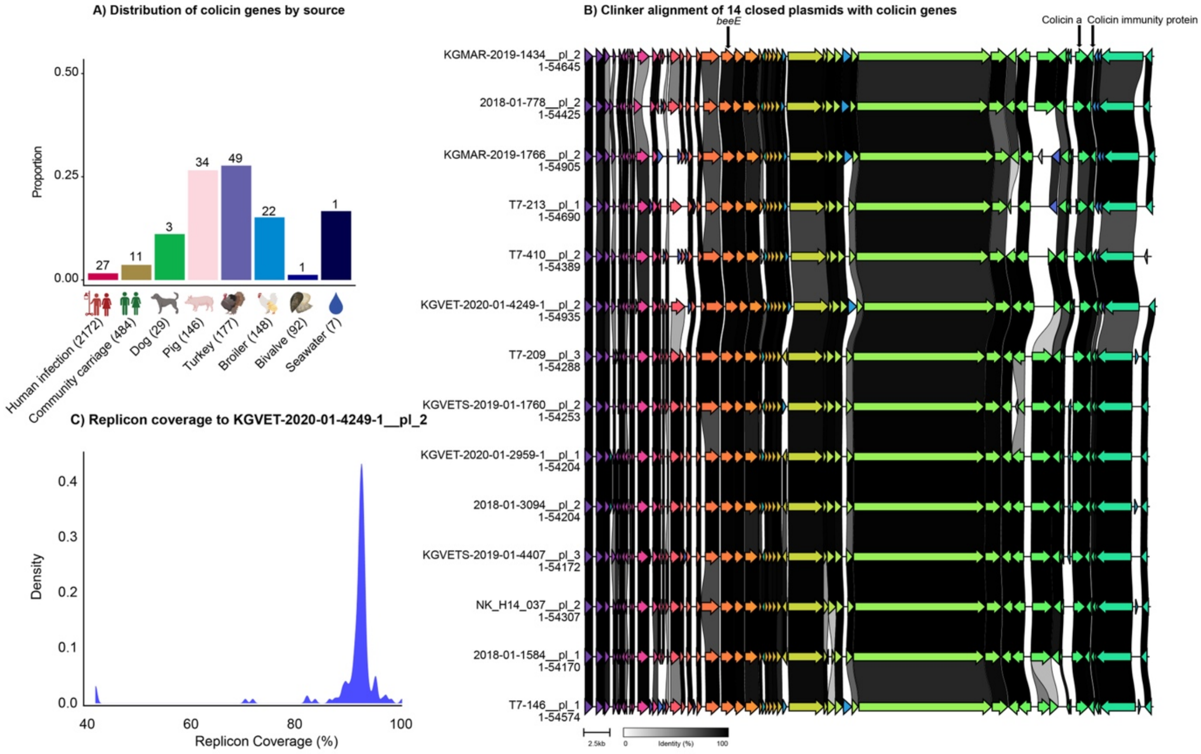

Fig. S12. Distribution of colicin genes. A) By source; colicin a and colicin immunity protein were co-distributed in 148 genomes across the eight sources. **B)** Clinker-alignment of closed replicons encoding colicin. Fourteen of the 148 genomes had closed genomes and were aligned, revealing that the colicin genes (indicated on top of plot) were located next to each other on highly similar plasmids. **C)** Genomes with the colicin-encoding plasmid. The largest plasmid from B) was used as a reference to align the 148 short-read sequenced genomes against, revealing that the colicin-genes were found on highly similar plasmids in most genomes.

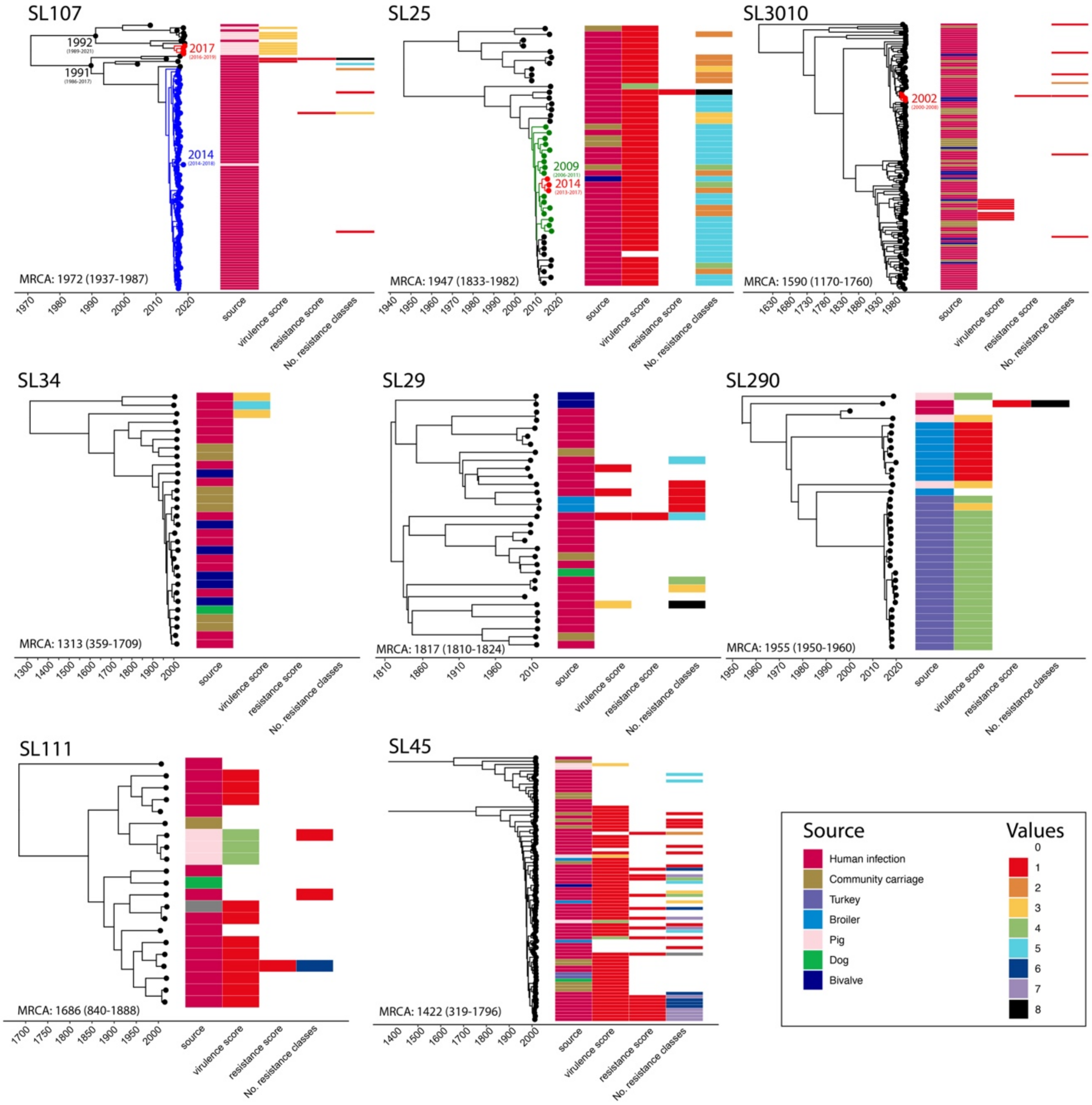

Fig. S13. Dated trees of prevalent niche-overlapping SLs. The most prevalent niche-overlapping SLs were old with variable AMR and virulence content. The dated phylogenies are shown together with source information, virulence score, resistance score and the number of antimicrobial resistance classes (from Kleborate v2.4.0). The most recent common ancestor (MRCA) of each SL is indicated with 95% HPD intervals. SL107, SL25 and SL3010 included cross-niche strain-sharing (shared ≤22 single nucleotide polymorphisms). The MRCAs of the genome pairs involved in those are indicated on the tips. The remaining dated SLs were frequent across the niches but did not include cross-niche strain-sharing at ≤22 SNP (see also Table S3).

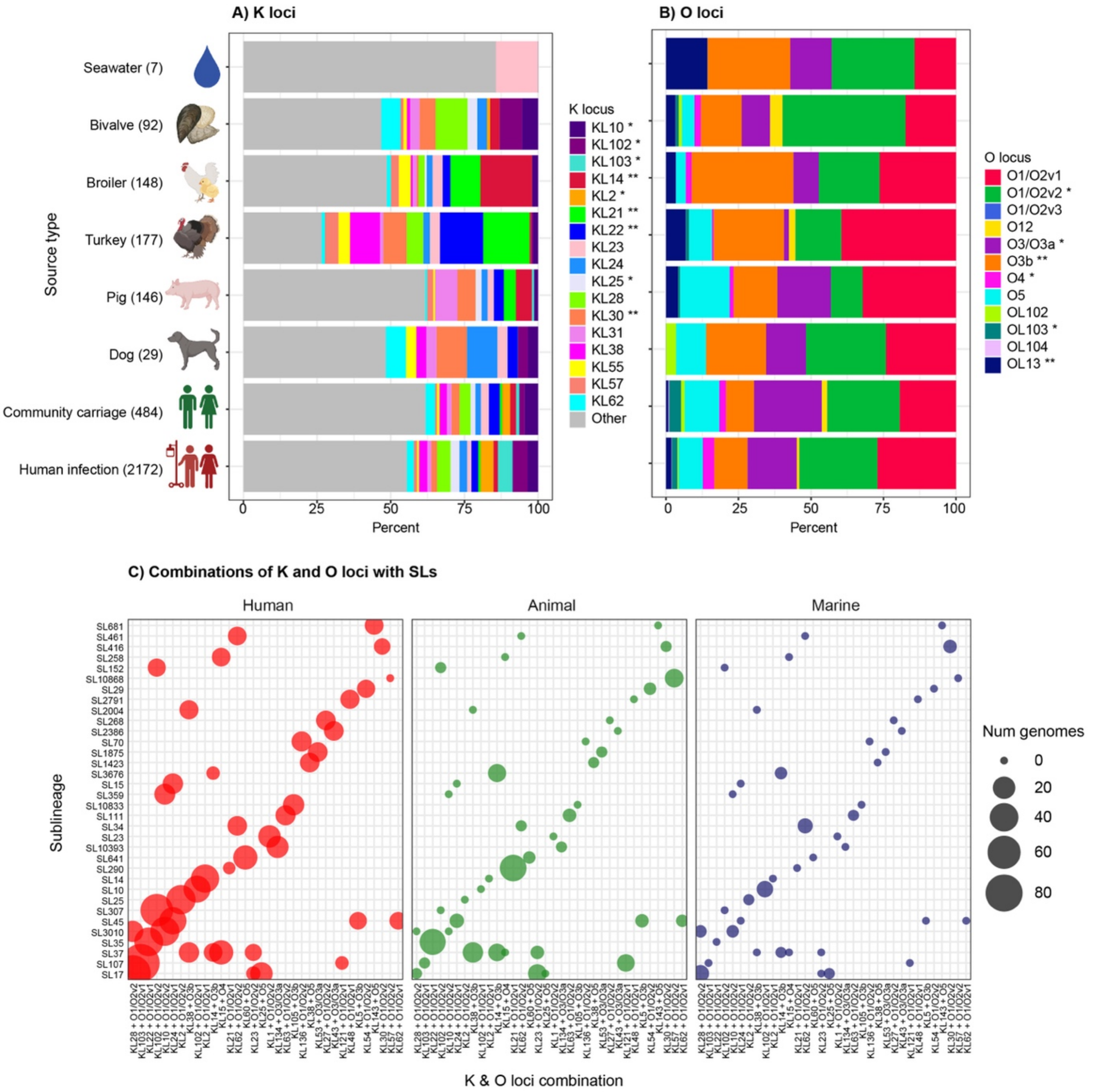

Fig. S14. Capsule (K) and O loci by source and sublineages (SLs). A) Distribution of K loci among the sources. The 10 most prevalent K loci from each of the human and non-human samples are shown. **B)** Distribution of O loci among the sources. * K and O loci that were overrepresented among the human samples; ** loci that were overrepresented in the non-human samples (see Table S7). **C)** The most common combinations (>10 genomes) of K and O loci (x-axis) and SLs (y-axis). The bubbles indicate the number of genomes, and are shown for each of the three niches: human, animal and marine.

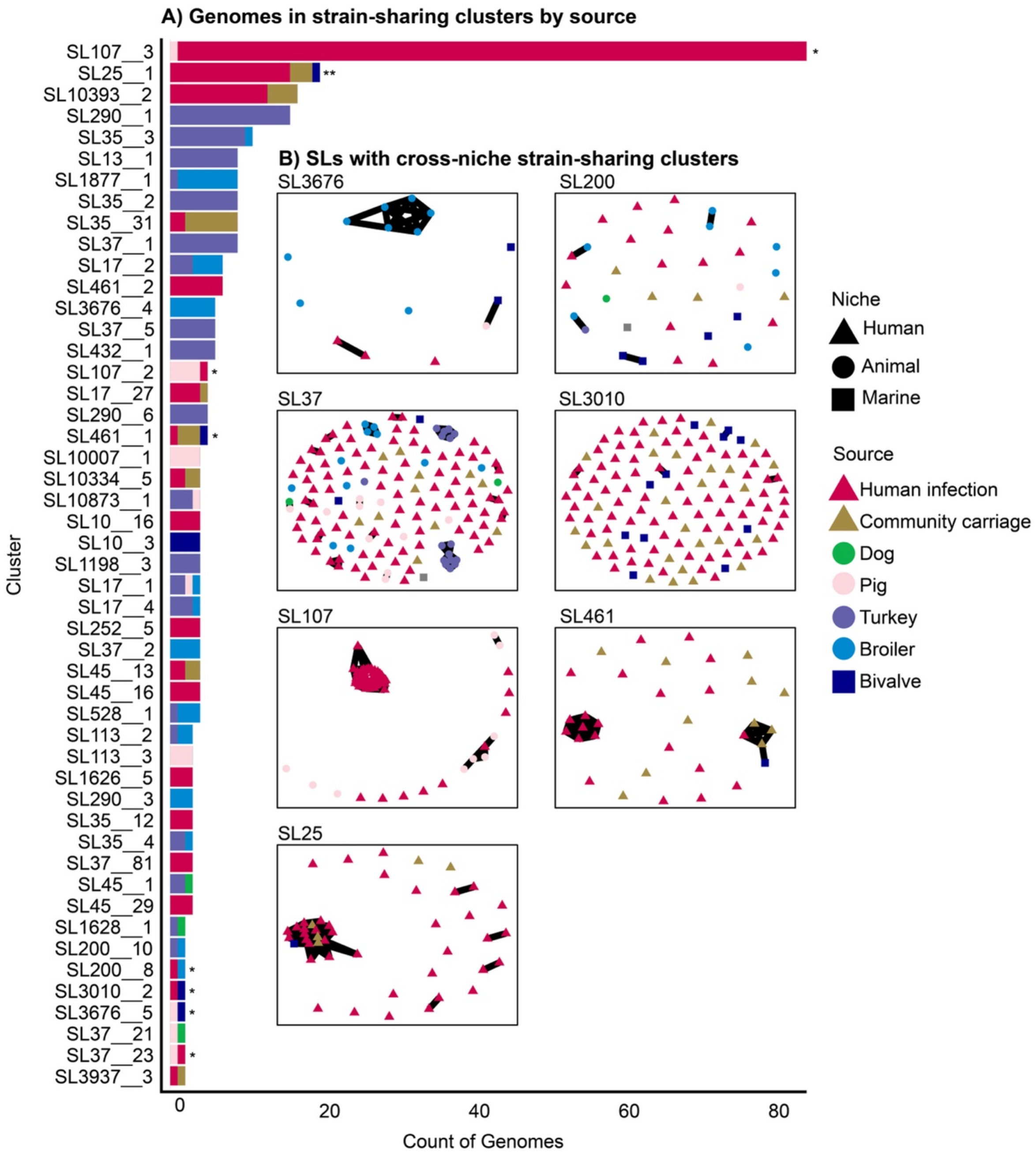

Fig. S15. Strain-sharing clusters by source. A) Number of genomes within strain-sharing clusters (sharing ≤22 SNPs), coloured by source. All strain-sharing pairs are shown, except for clusters with only 2 genomes where they came from the same source (62 human infection, 9 community carriage, 8 pig, 4 turkey, 9 broiler and 13 bivalve pairs). To distinguish clusters within the same SL, cluster names were assigned using the SL and a sequential number. **B)** Seven SLs had ≥1 strain-sharing cluster between ecological niches (indicated with * in A). The nodes represent the genomes within each SL. Lines were drawn between pairs of genomes if they shared ≤22 SNPs. The nodes are shaped by niche and coloured by source (inset legend).
